# A novel anti-melanoma SRC-family kinase inhibitor

**DOI:** 10.18632/oncotarget.26787

**Published:** 2019-03-19

**Authors:** Ruth Halaban, Antonella Bacchiocchi, Robert Straub, Jian Cao, Mario Sznol, Deepak Narayan, Ahmed Allam, Michael Krauthammer, Tarek S. Mansour

**Affiliations:** ^1^ Department of Dermatology, Yale University School of Medicine, New Haven, Connecticut, USA; ^2^ Department of Pathology, Yale University School of Medicine, New Haven, Connecticut, USA; ^3^ Comprehensive Cancer Center Section of Medical Oncology, Yale University School of Medicine, New Haven, Connecticut, USA; ^4^ Department of Surgery, Yale University School of Medicine, New Haven, Connecticut, USA; ^5^ Department of Quantitative Biomedicine, University of Zurich, Zurich, Switzerland; ^6^ Program in Computational Biology and Bioinformatics, Yale University School of Medicine, New Haven, Connecticut, USA; ^7^ Sabila Biosciences LLC, New City, New York, USA

**Keywords:** melanoma, SRC-family kinase inhibitors, MAPK, MITF

## Abstract

The major drawback of melanoma therapy with BRAF and MAPK inhibitors is the innate and acquired drug resistance. We therefore explored alternative targets and developed a new compound, SAB298, that is a SRC-family kinase (SFK) inhibitor. The drug is cytotoxic to patient-derived melanoma cells regardless of oncogene expression and inhibits tumor growth *in vivo*. As expected, it inhibited SRC and PI3K activity, and had the additional property of ERBB2 inhibition, that lead to inactivation of the two ERK phosphatases PP2A and SHP2. In 57% of the melanoma cell lines tested, the consequent increase in ERK activity lead to proteolytic degradation of its substrate, the lineage specific transcription factor MITF, likely contributing to growth arrest. Treatment with a combination of SAB298 and AZD6244 (selumetinib), induced a synergistic growth inhibition, suggesting that the new compound could be used in the clinic as a substitute for, or in combination with MAPK inhibitors.

## INTRODUCTION

Current standard therapies for melanoma include treatments with checkpoint blockades or with vemurafenib and dabrafenib, small molecule inhibitors for BRAF and MEK. The combination therapy diminishes the activity of BRAF^V600E/K^ present in 40–50% of melanomas [1, 2] and inhibits the mitogen-activated protein kinase (MAPK) pathway. However, there is an urgent need to identify new molecular pathways for targeting melanomas because of inherent or rapid emergence of resistance to MAPK inhibition [3–5]. In addition, melanomas that do not carry the BRAF oncogenes cannot be subjected to this type of therapy and are limited to treatments with immune checkpoint inhibitors, such as nivolumab, or combination of nivolumab with ipilimumab, approved by the FDA in 2016.

We sought to expand therapeutic possibilities using medicinal chemistry optimization strategies which led to the discovery of SAB298, a new kinase inhibitor that targets mostly members of the SRC-family kinase (SFK). SAB298 is an efficient inhibitor of a range of patient-derived melanoma cell lines, displaying a unique mechanism of action compared to other known SRC kinase inhibitors.

## RESULTS

### SAB298 is the most cytotoxic SFK inhibitor to melanoma cells

**Figure 1:**
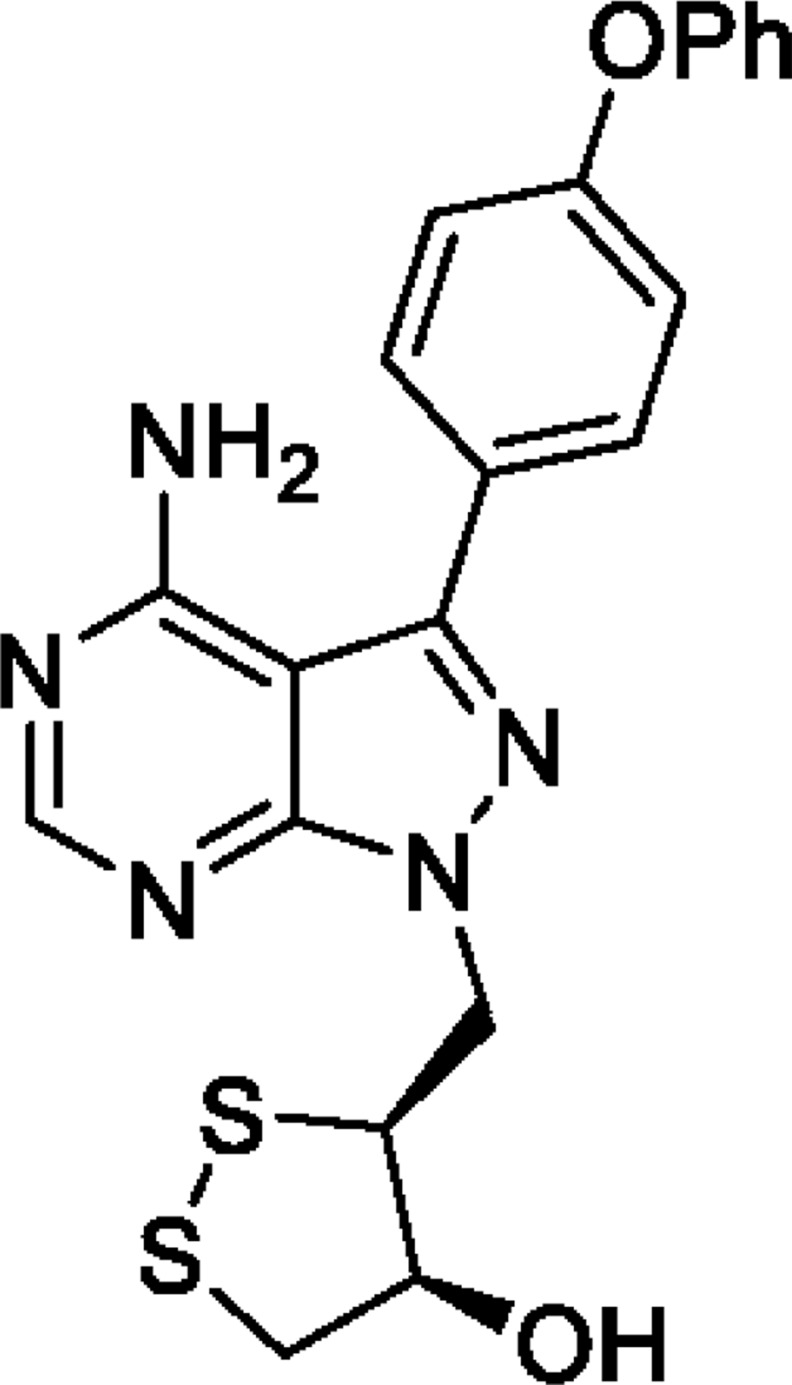
Structure of SAB298 The compound contains the pyrazolo[3,4-*d*]pyrimidine core structure, has the (3*S*, 4*S*) absolute configuration at the two stereogenic centers and features a substituted 1,2 dithiolane moiety that is unprecedented amongst kinase inhibitors. Its chemical name is (3*S*, 4*S*)-3-((4-amino-3-(4-phenoxyphenyl)-1*H*-pyrazolo[3,4-*d*]pyrimidin-1-yl)methyl)-1,2-dithiolan-4-ol.

SAB298 is a synthetic small molecule that has emerged from a rational design strategy for receptor and non-receptor tyrosine kinases’ inhibitor. It contains the pyrazolo[3,4-*d*]pyrimidine core structure, has the (3*S*, 4*S*) absolute configuration at the two stereogenic centers and features a substituted 1,2 dithiolane moiety that is unprecedented in kinase inhibitors (Figure 1).

**Figure 2:**
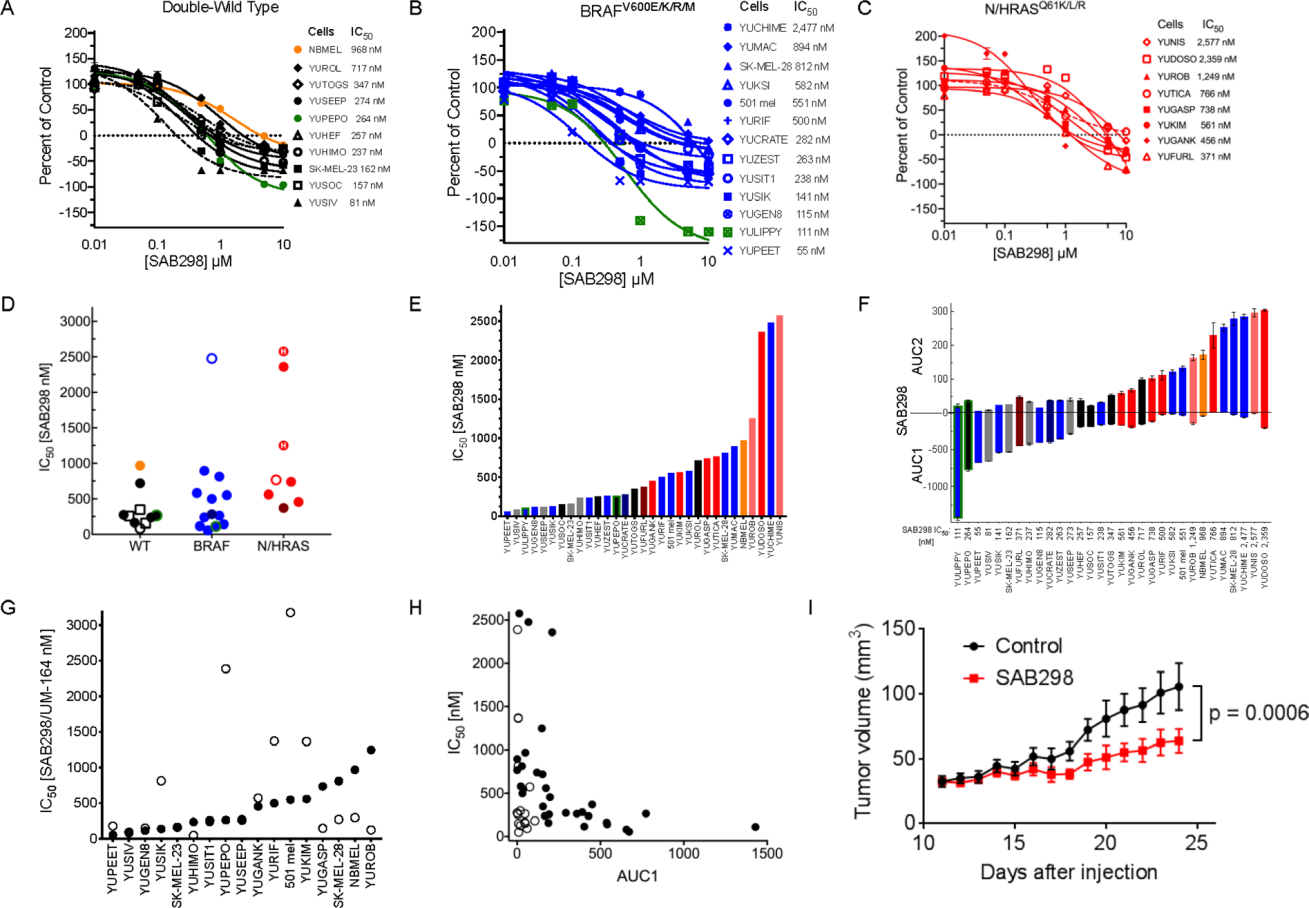
Suppression of cell proliferation in response to SAB298 and UM-164 The results show CellTiter-Glo^®^ Luminescent Viability Assay and the values are average of triplicate or quadruplet wells, displayed as percent of control assessed at the end of 72 hours treatment ± SE. (**A**–**C**) Growth arrest in response to SAB298. The legends on the right provides the IC_50_, i.e., drug concentrations that reduced cell viability to 50% of the control generated in GraphPad Prism. Black, blue and red lines indicate melanoma cells that are wild-type for BRAF or NRAS (double-wild type), BRAF^V600E/K^, or NRAS/HRAS^Q61^ mutants, respectively. Green lines indicate ocular melanoma cells with GNA11^Q209L^, broken lines are NF1^null^ (---)_,_ broken dot broken line are RAC1^P29S^/NF1^null^ double mutant (-.-), and orange line indicates normal human melanocytes that are wild type for all mutations. STDV was about 5% of total count. (**D**–**F**) show aligned dot-plot of SAB298 IC_50_ values, bar graph of increasing levels of IC_50_ and AUC (Area Under the Curve). Color code as in (**A**–**C**). Grey bars indicate melanoma with fusion genes and (H), indicates HRAS^Q61K^ mutation. (**G** and **H**) show comparisons between SAB298 (●) and UM-164 ○ in IC_50_, AUC1. (**I**) Tumor growth in response to SAB298. YUSIK tumor bearing mice were treated daily with intraperitoneal injection of 20 mg/kg SAB298 (red) starting on day 12 after injection or with solvent as control (blue). Data are average of 6 mice ± SEM. The *p*-value of 0.0006 was calculated by Uncorrected Fisher’s LSD using GraphPad based on the last day data. 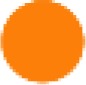 NBMEL; 
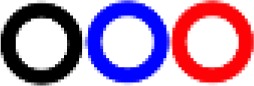
 NF1; 
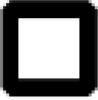
 NF1 and RAC1; 
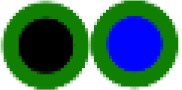
 GNA11^Q209L^; 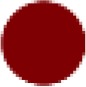 NRAS^G12D^; 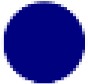 BRAF^G469A^.

We first tested the impact of SAB298 on NCI-60 human tumor cell lines that included eight melanomas (performed by the NCI Development Therapeutics Program). The results showed suppression of melanoma cell proliferation (IC_50_ of 21–550 nM), with the *BRAF*^*V600E*^ mutant melanoma cell line UCLA-SO-M14 (M14) being most sensitive (Supplementary Table 1). We therefore went on to screen the drug effect on a cohort of 30 patient-derived short-term cultures of melanoma cell lines carrying *BRAF*^*V600E/K*^, *NRAS*^*Q61K/L/*R^, *NF1*^*null*^, *RAC1*^*P129S*^, *RAF1* or *BRA*F fusion proteins, as well as other mutations identified by exome-capture sequencing (Table 1 and Supplementary Table 2). Overall, the drug was particularly cytotoxic to cell lines that did not carry oncogenic mutations in *BRAF* or *NRAS* (double-WT), displaying a range of IC_50_ between 81–717 nM (Median = 257 nM). Melanomas with *BRAF*^*V600E/*K^ sub-grouped into lines that were sensitive (IC_50_ 55–282, Median =141 nM) or resistant (IC_50_ 500–2,477 nM, Median = 697 nM), while the *NRAS*^*Q61K/L/R*^ mutants required relatively high concentrations of SAB298 to induce growth arrest (IC_50_ 371–3,080 nM, Median = 752 nM) (Figure 2A, 2B and 2C, summarized in 2D–2F). Interestingly, normal human melanocytes grouped with the resistant subtype (IC_50_ 968 nM, Figure 2A, orange). The cytotoxicity of SAB298 was well demonstrated by the high values of AUC (Area Under the Curve) below the zero line for double wild-type melanoma cell lines (Figure 2F). Three-way Kruskal-Wallis analysis of the dot-plot display (Figure 2D) showed that the difference in the levels of response to SAB298 between the double-WT and NRAS-mutant melanoma cells were statistically significant, *p*-value = 0.002985.

**Table 1:** Melanoma cell lines

Melanoma type	Number of specimens
Sun-exposed	17
Acral	5
Ocular	2
Mucosal	1
Unknown	5
**Total**	**30**
BRAF/RAS Mutations^*^	**Number of Specimens**
WT/WT	10
BRAF^V600E/K/M^	10
NRAS/HRAS^Q61K/R,G12D^	9
BRAF^G469A^	1

^*^See Supplementary Table 2 for more information regarding the presence of other cancer genes.

SAB298 (0.5 µM and 1 µM) induced apoptosis as measured by annexin staining and caspase activity (Supplementary Figure 1A–1C).

Comparing the potency of SAB298 to clinically-relevant SFK-inhibitors showed that dasatinib, bosutinib, saracatinib, SU6656 and imatinib (the latter targeting ABL, KIT and PDGFR), had very little or no inhibitory effect (IC_50_ ∼10,000 nM, Supplementary Figure 2A). On the other hand, UM-164, a modified dasatinib that binds to the inactive conformation of SRC kinase Asp-Phe-Gly motif (DFG-out) and inhibits also p38 kinase [6], was effective independent of oncogenic mutations (IC_50_ 50–3,000 nM) (Supplementary Figure 2B–2E). However, compared to SAB298, UM-164 was less effective inhibitor for ∼50% of the melanoma cell lines as indicated by the IC_50_ plots (Figure 2G), and less cytotoxic, as shown by the low levels of AUC1 scores (Figure 2H, compare empty to solid dots, and supplementary Figure 2E). We also confirmed the anti-melanoma activity of SAB298 *in vivo*. Daily administration (20 mg/kg), significantly suppressed the growth of established YUSIK melanoma tumors, compared to placebo control (*p* = 0.0006) (Figure 2I).

**Figure 3:**
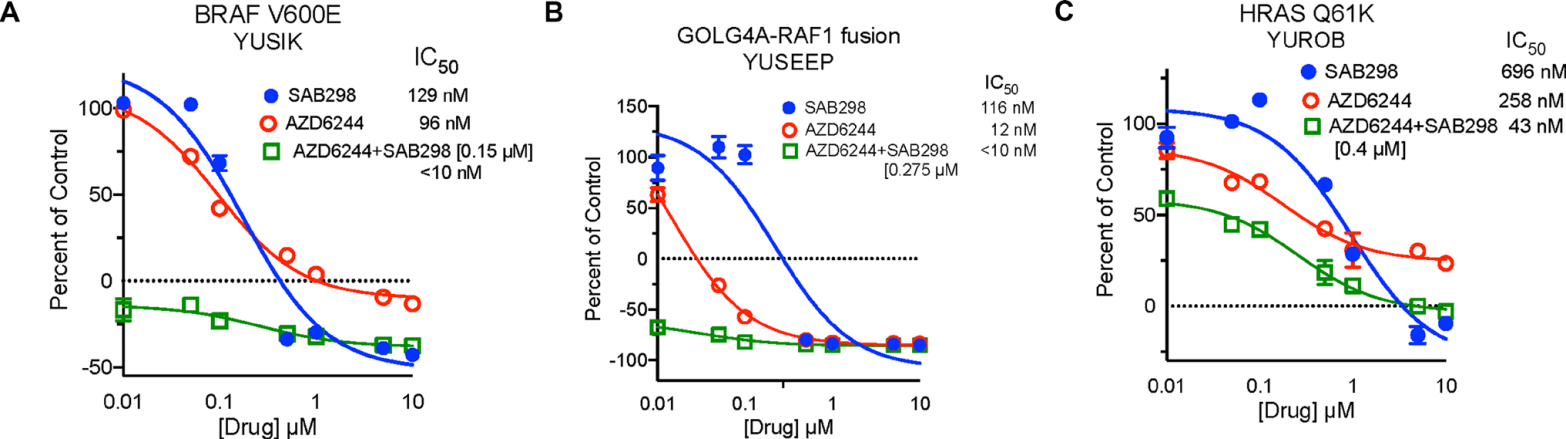
Synergistic Inhibition of BRAF^V600E^ Melanoma Cell Proliferation by Treatment with SFK and MEK Inhibitors Cell proliferation of YUSIK, 501 mel and YUROB melanoma cell lines (**A**, **B** and **C**, respectively) in response to SAB298 (blue), AZD6244 (red), and AZD6244 plus SAB298 (green). SAB298 was added to AZD6244 at 0.15 µM (YUSIK), or 0.4 µM (501 mel and YUROB). The IC_50_ for each drug alone and in combination are indicated in the legend for each cell line.

SAB298 suppressed cell proliferation as effectively as the ERK and MEK inhibitors (SCH772984 and AZD6244, respectively), which displayed wide range of activity when tested on our cohort of melanoma cells (IC_50_ 12->10,000 nM) [7]. In addition, SAB298 had synergistic inhibitory effect when combined with the MEK inhibitor AZD6244. The combination treatment dropped the IC_50_ values to <10 nM, 127 nM and 43 nM in the BRAF^V600E^ mutants YUSIK, 501 mel cells and the HRAS^Q61K^ mutant YUROB cells, respectively (Figure 3). The combination treatment had statistically strong synergism assuming mutually exclusive and nonexclusive mode of action of AZD6244 and SAB298 across all three cell lines. The Combination Index (CI) values were between 0.1 and 0.3 based on the median-effect equation derived from the mass-action law principle [8, 9] to quantify synergism (see Supplementary Methods for details).

Altogether, the results show that SAB298 is an effective inhibitor of melanoma cell proliferation and can be used for targeted therapy in melanoma.

### SAB298 inhibits SRC-family kinases, ERBB2 and PIK3R

We performed radioisotope filtration binding assay [10] that showed high affinity of SAB298 to several SRC-family kinases (SFK), such as YES1, BLK, LCK, FGR, HCK and FYN (IC_50_ ranging from 0.7–21.7 nM), but very little to BRAF, RAF1, ARAF, ABL1, ABL2, WEE1 and ERBB2 (IC_50_ 1,200->10,000 nM), or to IGF1R and CDK4/cyclin D1 (IC_50_ >10,000 nM) (Supplementary Table 3). Furthermore, we applied the *in situ* KiNativ^®^ screening test that confirmed the kinase selectivity of SAB298 against several SFKs (IC_50_ <10–60 nM) including SRC, YES1, LYN, and CSK, and against ABL2, and the receptor kinases ERBB2 and ERBB3, with some variability between the two cell lines tested (YUSIV and YUSIK, Table 2). The ∼10-fold and ∼20-fold higher binding affinity of SAB298 to SRC and ERBB2 in the *in situ* KiNativ^®^ assay compared to the radioisotope filtration binding assay done on purified compounds indicated stronger activity with the native conformation in the cellular milieu. There was very little activity against mTOR, EGFR, LCK and MEK1 (IC_50_ 1,300–2,900 nM, Table 2).

**Table 2:** KiNativ™ assay tests for SAB298 target protein^*^

Kinase	YUSIV	YUSIK
YES1	<10	Not Present
SRC	31	Not Present
LYN	60	190
ABL2	87	240
ERBB3	290	240
MEK5	370	1500
CSK	420	910
ERBB2	410	530
mTOR	1,300	2,000
EGFR	1,300	530
LCK	1,400	Not Present
RIPK2	Not Present	20
MEK1	2,900	Not Present

^*^The results show IC_50_ in nM. Values were determined from 3-point dose response curve.

**Figure 4:**
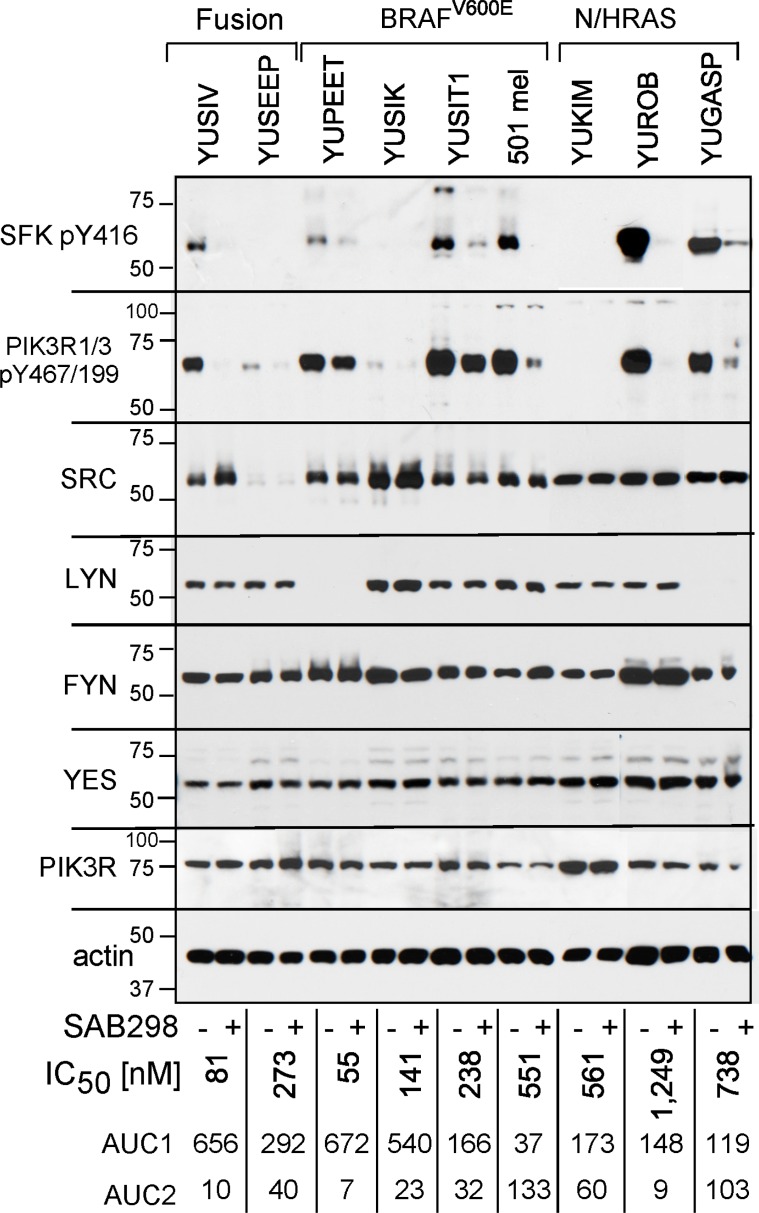
SAB298 suppresses SFK and PI3K activities Western blot analyses of melanoma cells treated with DMSO or SAB298 (0.5 µM) for 5 hrs. Cell extracts were probed with antibodies to phosphorylated SFKs (SFK pY416), phosphorylated PI3KR1/3 (pY467/199), to different SFKs or PIK3R as indicated. Reduction in the levels of pSFK Y416 in response to SAB298 indicates inhibition of SFK. Two cell lines (SK-MEL-28 and YUSEEP) did not express detectable levels of SFK pY416. Anti-actin represents protein loading. The levels of growth responses to SAB298 are indicated by the IC_50_, AUC1 and AUC2 on the bottom of each lane. YUSIV and YUSEEP carry PDE8A-RAF1 or GOLGA4-RAF1 fusion proteins, respectively.

We confirmed that SAB298 inhibits SFK by probing with anti-IEDNEpYTAR antibodies for auto-phosphorylated Y416 (pY416). Melanoma cell lines displayed variable levels of basal pY416, which was consistently suppressed by SAB298 (Figure 4, pY416 SFK). In addition, SAB298 suppressed PI3K, the SRC downstream signaling target [11] observed by decrease in phospho-Y467/199 PIK3R1/3 (Figure 4, pY467/199 PIK3R1/3). Interestingly, there was a correlation between the levels of pY416 SFK and pY467/199 PIK3R; cells that had very low or undetectable levels of pY416 SFK also displayed low or undetectable levels of pY467/199 PIK3R (Figure 4, YUSEEP, YUSIK and YUKIM). However, the basal levels of pY416 SFK or pY467/199 PIK3R were not markers for the sensitivity to the compound (Figure 4, compare the highly resistant 501 mel, YUGASP and YUROB to the sensitive YUSIV, YUSIK and YUSEEP, IC_50_ and AUC shown on the bottom of each lane). In addition, the cell lines expressed different levels of SFK members, SRC, LYN, FYN and YES (Figure 4, as indicated), that may have a role in drug response.

**Figure 5:**
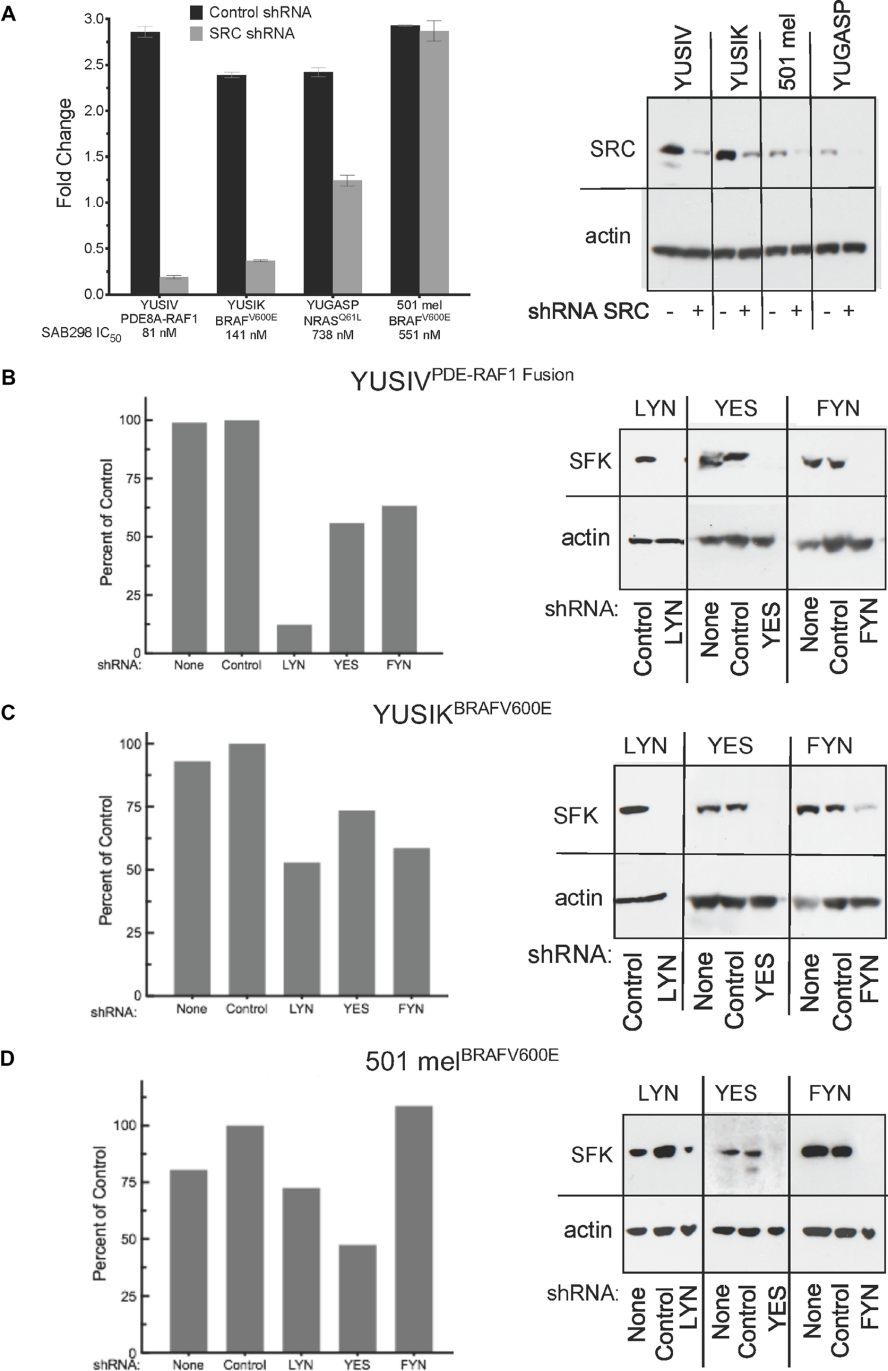
Cell proliferation in response to downregulation of specific SFKs The histograms show cell proliferation of melanoma cells infected with shRNA targeting SRC, LYN, YES or FYN compared to none-infected cells (None) and control shRNA (Control) (left panels **A**–**D**). The shRNA knockdown was validated by western probing for the expression of the SFK employing the respective antibodies (right panels A–D). SFK: SRC Family Kinase.

We performed short hairpin RNA (shRNA) knockdown of SRC, LYN, YES, and FYN to identify the “addictive” SFK (Figure 5). The results show that SRC depletion in YUSIV, YUSIK and YUGASP melanoma cells induced growth arrest whereas 501 mel cells were not affected (Figure 5A). YUSIV melanoma cell line, the most sensitive to inhibition by SAB298, required LYN for optimal cell proliferation (Figure 5B), whereas there was only at most 50% reduction in growth in response to downregulation of YES and FYN (Figure 5B, 5C). Here again, the most resistant were 501 mel melanoma cells (Figure 5D).

### SAB298 stimulates rather than inhibits MAPK signaling

**Figure 6:**
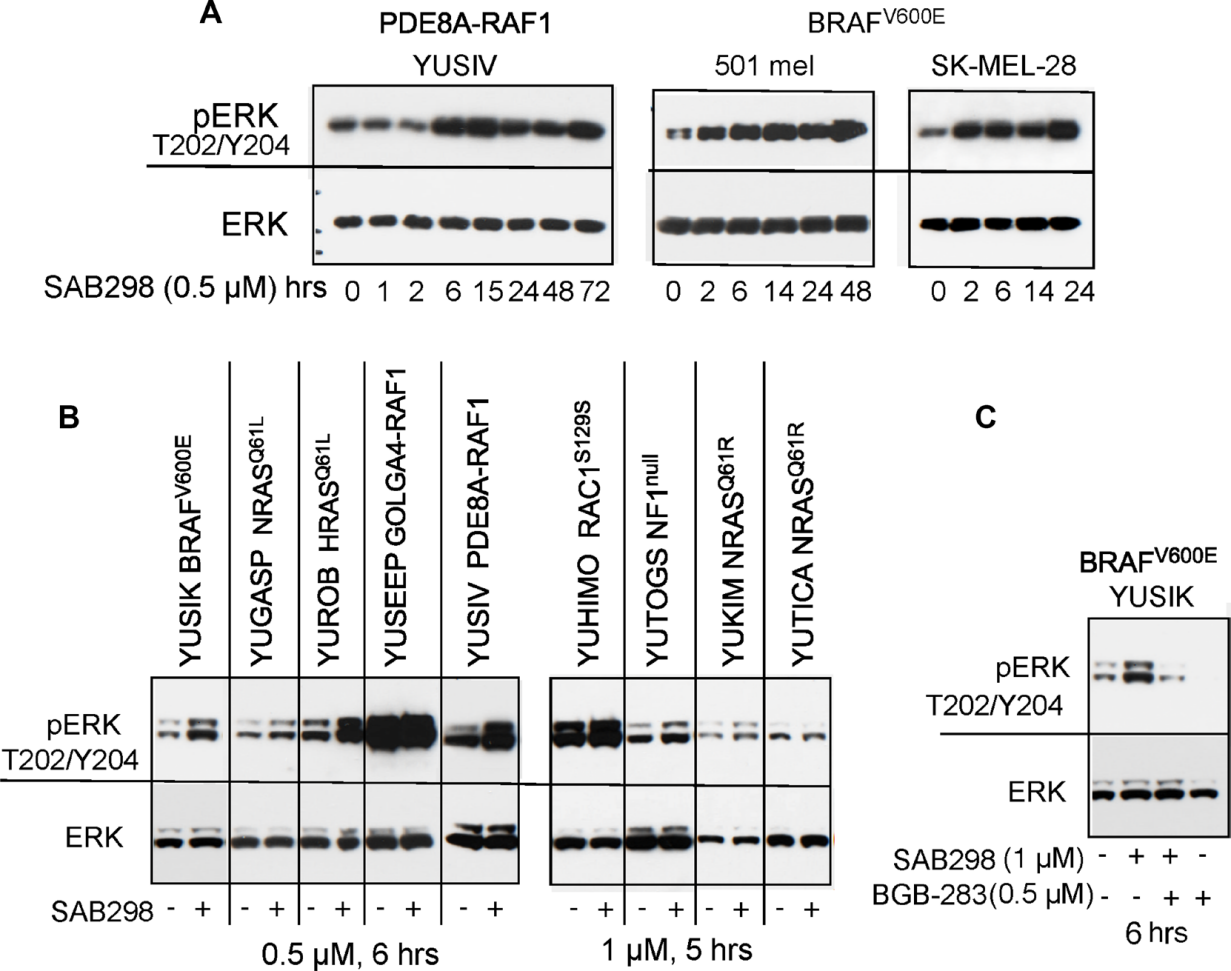
MAPK signaling is activated in response to SAB298 Melanoma cells were treated with SAB298 (0.5 µM or 1 µM, as indicated) and harvested at increasing time points (**A**), or after 5-6 hours (**B**, **C**). The panels show western blots probed with antibodies to phosph-ERK1/2 Thr202/Tyr204 mAb (pERK), ERK1/2 (ERK), phospho-MEK1/2 (pMEK), and actin as protein loading control. (**C**) Cells were treated with SAB298, BGB-283 (a RAF-dimer inhibitor), or both for 6 hrs to validate the role of RAF kinase in ERK phosphorylation. The mutation status of each cell line is indicated on the top.

**Figure 7:**
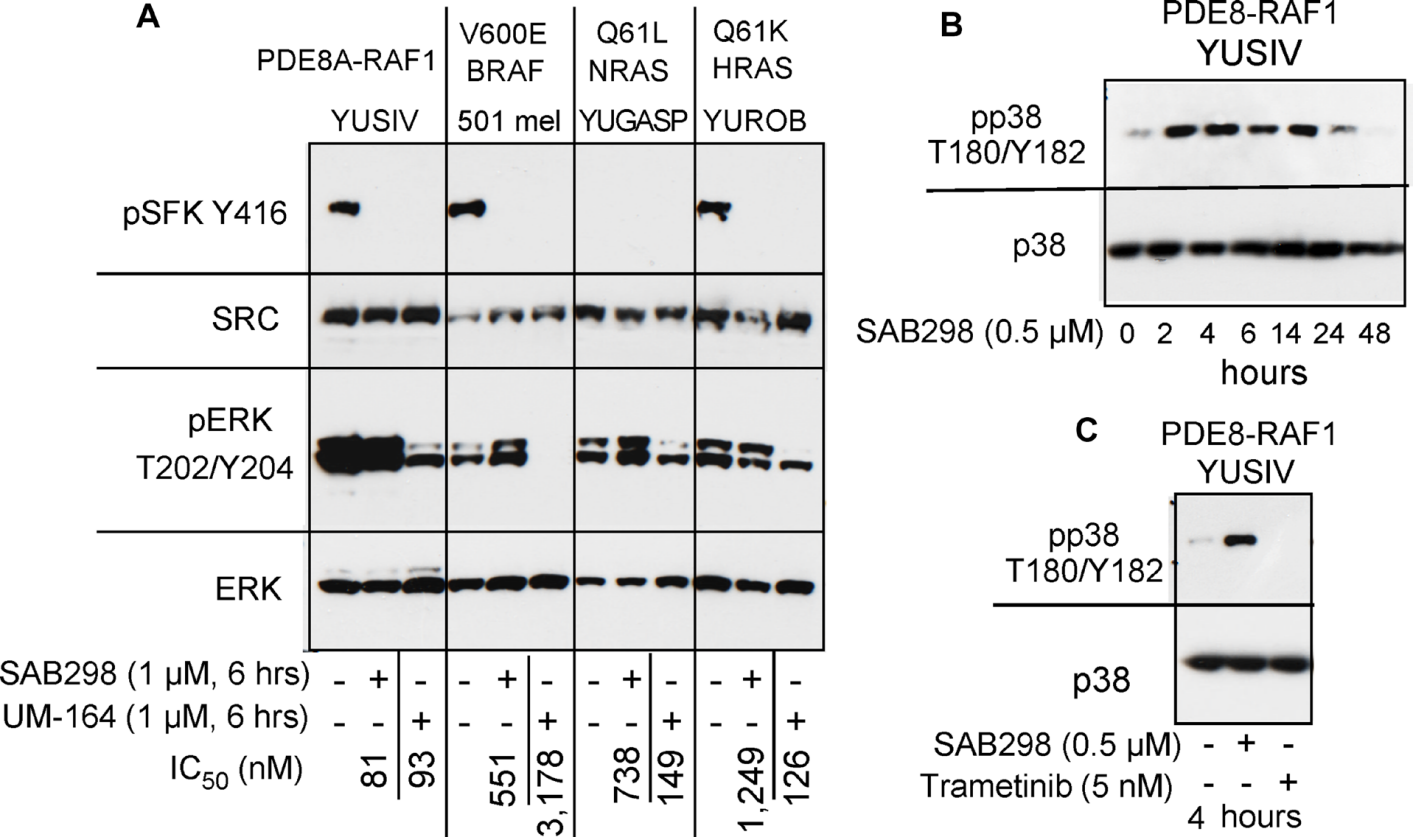
SAB298 and UM-164 inhibit SFK activity but have opposite effects on ERK and p38 (**A**) Western blot showing common inhibition of SFK (pSFK Y416) in YUSIV, 501 mel and YUROB melanoma cells in response to the two inhibitors, but pERK was inhibited only in response to UM-164 (pERK T202/Y204). (**B**) stimulation of pp38 T180/Y182 in YUSIV melanoma cells incubated for increasing periods of time with SAB298. (**C**) Trametinib, but not SAB298, inhibits pp38 T180/Y182.

Time-course analysis revealed that treatment with SAB298 increased, rather than decreased, the levels of phospho-MEK S217/221 and phospho-ERK S217/221 (pERK T202/Y204). This effect was observed within 2–6 hours and was maintained throughout the 72 hours incubation with the inhibitor (Figure 6A). This unexpected long term signaling response was observed in YUSIV that carries PDE8A-RAF1 fusion protein and in *BRAF*^V600E^ mutant melanomas (501 mel and SK-MEL-28). A similar response was observed in seven additional melanoma cell lines regardless of the presence or absence of *BRAF* or *NRAS* oncogenic mutations (Figure 6B). In all cases, there was either an increase in pERK Thr202/Tyr204 or no effect, especially when the levels of basal pERK Thr202/Tyr204 were high (Figure 6B and Figure 7A). RAF activity was required to maintain pERK because the RAF1-dimer inhibitor BGB-283 abolished pERK (Figure 6C).

We examined if the “paradoxical” activation of the MAPK pathway is a common response to SRC inhibition in these melanoma cells. Side-by-side comparison of SAB298 and UM-164 confirmed that the two inhibitors downregulated pSFK Y416, but had an opposite effect on pERK T202/Y204 (Figure 7A). Additionally, in contrast to UM-164, SAB298 induced p38 T180/Y182 phosphorylation within two hours, in a manner similar to that of pERK T202/Y204 stimulation (compare Figure 7B to Figure 6A), but this effect subsided after ∼24 hrs (Figure 7B). The results were not due to aberrant p38 response in these melanoma cells, because the MEK1/2 inhibitor trametinib effectively abolished pp38 T180/Y182 (Figure 7C). The p38 kinase inhibitors ralimetinib and LY2228820 had little effect on melanoma cells (IC_50_ >10,000 nM data not shown), suggesting that this kinase does not carry a critical role in melanoma cell proliferation as it does for triple-negative breast cancer cells [6]. Altogether, we show that the SFK-inhibitor SAB298 is a potent suppressor of melanoma cell proliferation independent of pERK activity.

### SAB298 inactivates MAPK phosphatases via inhibition of ERBB2

**Figure 8:**
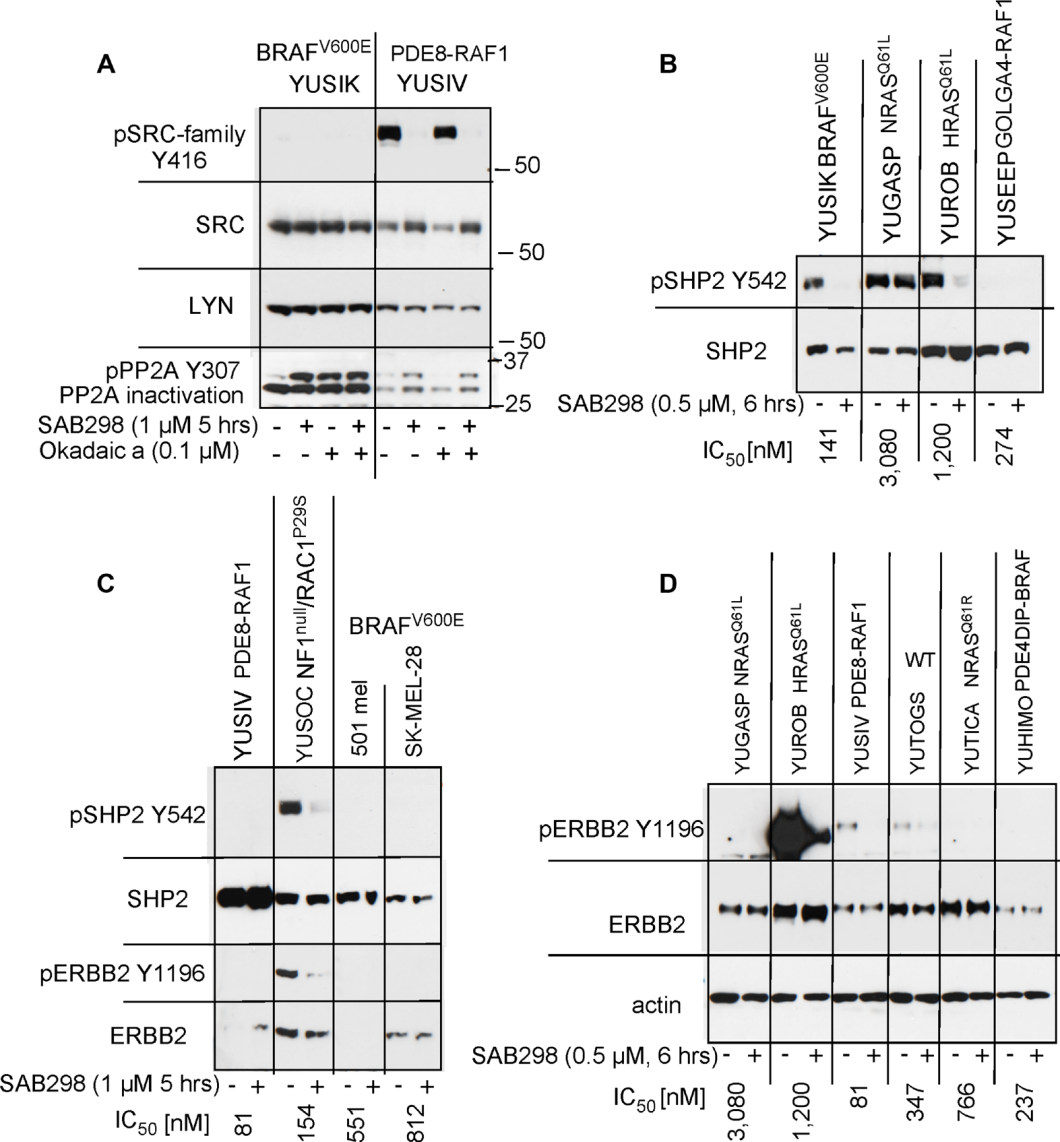
SAB298 inactivates PP2A, SHP2 via ERBB2 inhibition (**A**) SAB298 increases the levels of inactivated PP2A (pPP2A Y307) in YUSIK and YUSIV melanoma cells in a fashion similar to okadaic acid (Okadaic a). Active (unphosphorylated) PP2A is not dependent on the presence of pY416 SFK. Panels **B** and **C** show SHP2 inactivation by SAB298 in YUSIK, YUROB and YUSOC, but not YUGASP melanoma cells (pSHP2 Y542). Suppression of SHP2 is associated with SAB298 induced inactivation of ERBB2 (pERBB2 Y1196) (**C**, **D**).

The SAB298 induced MAPK activation was not the direct effect of SFK inhibition because it was not observed in response to knockdown of SRC, YES or FYN (Supplementary Figure 3). We therefore explored the role of two MAPK phosphatases, PP2A (encoded by *PPP2CA*) and SHP2 (encoded by *PTPN11*) [12–14]. Probing with antibodies to the inactivated PP2A (phospho-PP2A Y307) [15], showed increase in the levels of inactivated PP2A levels in response to SAB298, in a way similar to that of okadaic acid, a potent inhibitor of these protein phosphatases (Figure 8A, pPP2A Y307). In addition, SAB298 inactivated phospho-SHP2 Y542 in YUSIK, YUROB and YUSOC melanoma cell lines, but not in YUGASP (Figure 8B, 8C). The general levels of pSHP2 Y542 was inversely proportional to the levels of pERK (compare Figure 8 to Figure 6B).

Because SHP2 activity is modulated by receptor tyrosine kinases [16], we tested if ERBB2 inhibition, an additional *in situ* target of SAB298 (Table 2), is the cause of this process. The results confirmed that SAB298 suppressed phospho-ERBB2 Y1196 (Figure 8C and 8D, YUSOC, YUROB, YUSIV, YUTOGS). Interestingly, YUROB melanoma displayed extremely high levels of pERBB2 Y1196 that was abolished by SAB298 (Figure 8D). We concluded that pERK activation is the consequence of reduced PP2A and SHP2 activities, due to ERBB2 inhibition by SAB298. However, inhibition of ERBB2 with the potent ERBB2/ERBB3 inhibitor sapitinib, or with anti-ERBB3 antibody MM121, had very little effect on cell proliferation (YUSIV and YUSIK, IC_50_ >10,000 nM), while the EGFR/ ERBB2/ ERBB4 inhibitor dacomitinib had somewhat better inhibitory effect (IC_50_ 1,200 nM and 2,600 nM, respectively), suggesting that ERBB2 is not directly involved in SAB298 induced growth arrest.

### SAB298 downregulates MITF

**Figure 9:**
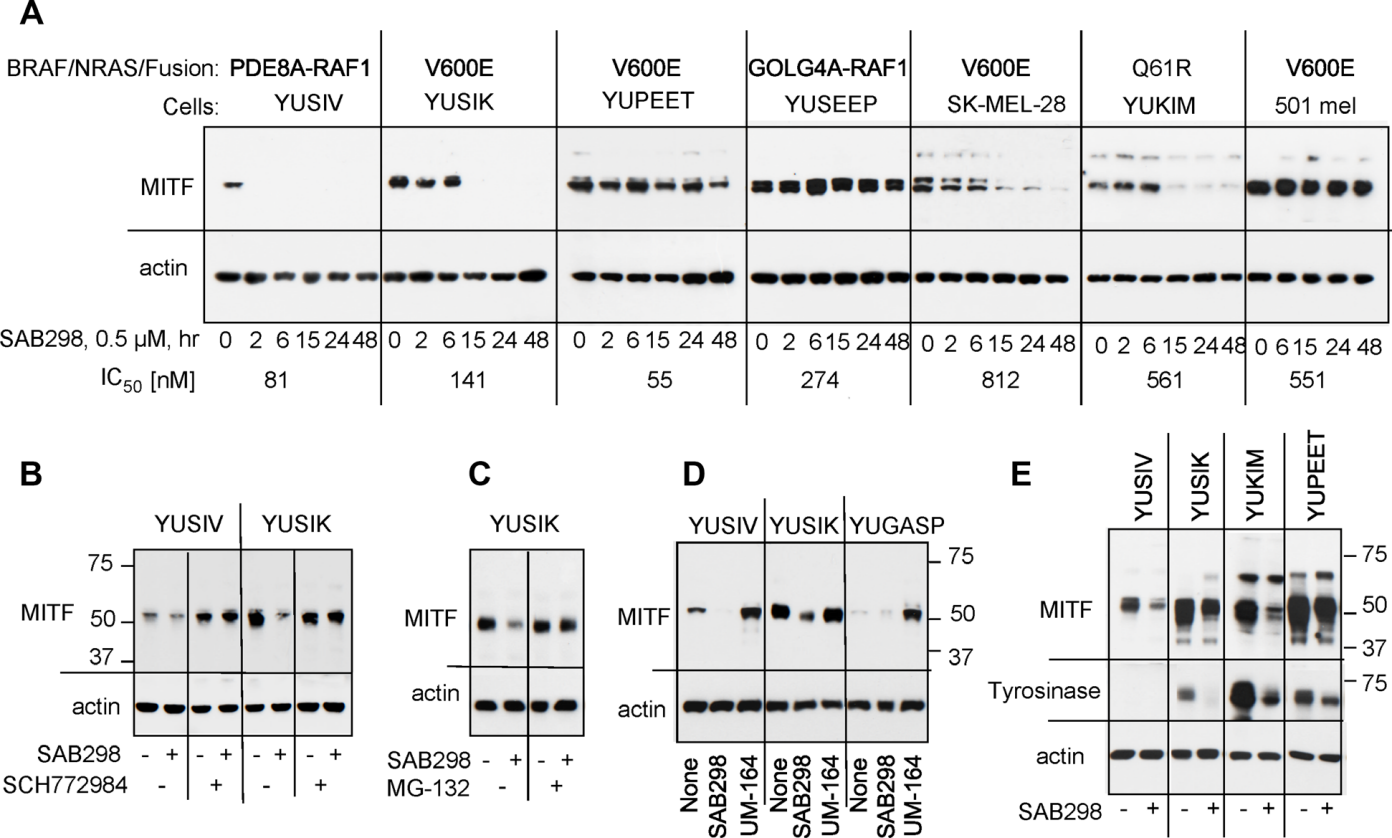
Suppression of MITF in response to SAB298 (**A**) MITF levels in melanoma cells incubated for increasing periods of times with SAB298 (0.5 µM). The oncogenic status of each cell line is indicated on the top and the IC_50_ on the bottom of the panels. (**B**) The MEK inhibitor SCH772984 (0.5 µM) abolished SAB298 (0.5 µM) induced suppression of MITF. Cells were harvested after 6 hrs treatment with the drugs. (**C**) The proteasomal inhibitor MG-132 (20 µM, 24 hrs) suppressed MITF degradation in response to SAB298. (**D**) The SRC inhibitor UM-164 (1 µM, 6 hrs) increases MITF expression. (**E**) Tyrosinase levels decrease in response to SAB298 (0.5 µM for 20 hrs) due to downregulation of MITF.

We examined the long-term effects of SAB298, such as the expression of cyclin D1, MYC, p27^CIP^, and p53, but did not observe any consistent shared response (data not shown). On the other hand, we checked the impact of ERK activation on MITF (melanogenesis associated transcription factor) because MITF is a critical transcription factor for melanocyte and melanoma cell proliferation [17], whose stability is reduced when phosphorylated by MAPK or KIT [18, 19]. The results showed that SAB298 suppressed MITF levels in four out of seven melanoma cell lines (YUSIV, YUSIK, SK-MEL-28 and YUKIM), but not in YUPEET, YUSEEP and 501 mel cells (Figure 9A). However, there was no correlation between IC_50_ levels and MITF downregulation (Figure 9A, indicated on the bottom for each cell line), suggesting that melanoma cells differ in their dependence on MITF.

The ERK-inhibitor SCH772984 validated the role of the kinase activity in downregulating MITF. Incubation with SCH772984 increased the basal levels of the transcription factor in YUSIV and abolished its downregulation by SAB298 in both YUSIV and YUSIK melanoma cells (Figure 9B). Furthermore, the addition of the proteasome inhibitor MG-132 (20 µM for 24 hrs) abolished SAB298 impact on MITF (Figure 9C), in agreement with published reports showing that the phosphorylated transcription factor is a target to proteasome degradation [18, 19]. In contrast to SAB298, UM-164 increased MITF levels (Figure 9D) as a consequence of ERK inhibition (Figure 7A).

We tested if modulation of MITF had a physiological effect as it can impact the expression of melanocyte-specific genes, such as tyrosinase. We show that indeed, SAB298 suppressed the levels of tyrosinase in melanoma cells that express the protein (Figure 9E, YUSIK, YUKIM and YUPEET), suggesting that downregulation of this transcription factor can contribute to SAB298 inhibition of cell proliferation in some cell lines.

## DISCUSSION

Targeting SRC in melanoma has been of interest for over a decade [20]. Published results with three melanoma cell lines showed that dasatinib and bosutinib had a minor impact (IC_50_ 1,300–10,000 nM) [20], and in another case, dasatinib did not have an effect on cell proliferation, but inhibited migration and invasion [21]. Additional studies showed that SRC-I1 (SRC inhibitor-1) was inactive on patient-derived BRAF^V600E^ mutant melanoma cells resistant to vemurafenib, but enhanced the activity of the pan-RAF inhibitor TAK632 against these cells [22]. Furthermore, phase 2 clinical trials with dasatinib and saracatinib had minimal clinical activity as a single agent in patients with advanced melanoma [23], and response rate to dasatinib among melanoma patients with KIT activating mutation was low [24]. This is in contrast to the highly efficient effect of dasatinib in BCR-ABL-driven diseases such as chronic myeloid leukemia (CML) and Philadelphia-chromosome-positive acute lymphoblastic leukemia (Ph+ ALL), characterized by the constitutively active tyrosine kinase, BCR-ABL [25].

We described a new compound, SAB298, that binds to the ATP kinase domain of several SFKs, and inhibits melanoma cell proliferation *in vitro* and *in vivo*, and affects other cancer cell types as well (lymphoblastic leukemia, carcinoma, astrocytoma) regardless of oncogene expression. The compound has multiple targets, the SFK family members and ERBB2/3, but has very little activity against BRAF, RAF1, ARAF, IGF1R or CDK4/Cyclin D1. It is common for kinase inhibitors to target more than one protein. For example, dasatinib inhibits SRC, c-Kit, ephrin receptors and BCR/Abl; imatinib targets ABL, KIT and PDGFR; SU6656 targets SRC family kinases and BRSK2, AMPK, Aurora C, Aurora B, CaMKKβ; and bosutinib and saracatinib inhibit SRC and ABL kinases. Quantitative analysis of 178 commercially available kinase inhibitors against a panel of 300 recombinant protein kinases revealed a wide spectrum of promiscuity and identified multitargeted inhibitors of specific, diverse kinases [26], indicating that SAB298 is not more promiscuous compared to other kinase inhibitors.

Our studies included 30 different patient-derived melanoma cell lines well characterized for mutations and genomic aberrations. Although double-wild type (BRAF/NRAS) melanoma cells were the most sensitive to the compound (with IC_50_ below 400 nM), the BRAF^V600^ mutant cells included a group of highly responsive and less responsive melanoma cells (IC_50_ below 400 nM and above 500 nM, respectively). Melanoma cells with oncogenic NRAS were the least sensitive (IC_50_ above 400 nM). On the other hand, alterations in NF1, including early termination, were not sufficient to raise the resistance of double-wild cells, such as YUSOC (IC_50_ 157), YUHEF (IC_50_ 257, NF1 pQ853X) and YUTOGS (IC_50_ 347, NF1 p.W336X/E337K), or cells carrying the fusion protein PDE8A-RAF1 YUSIV (IC_50_ 81 nM, NF1 p.L626F), but may had an impact on one BRAF^V600K/M^ melanoma (YUCHIME) displaying extreme resistance (IC_50_ 2,477 nM, NF1 p.K1714N). There was high correlation between the levels of pSFK and pPIK3R1/3, but the variability between the cellular responses did not correlate with the phosphorylated levels of these markers, and pSFK intensity levels did not correlate with the presence or absence of a melanoma oncogene. Interestingly, knockdown expression revealed that melanoma cells sensitive to the compound are “addicted” to SRC and LYN activity.

**Figure 10:**
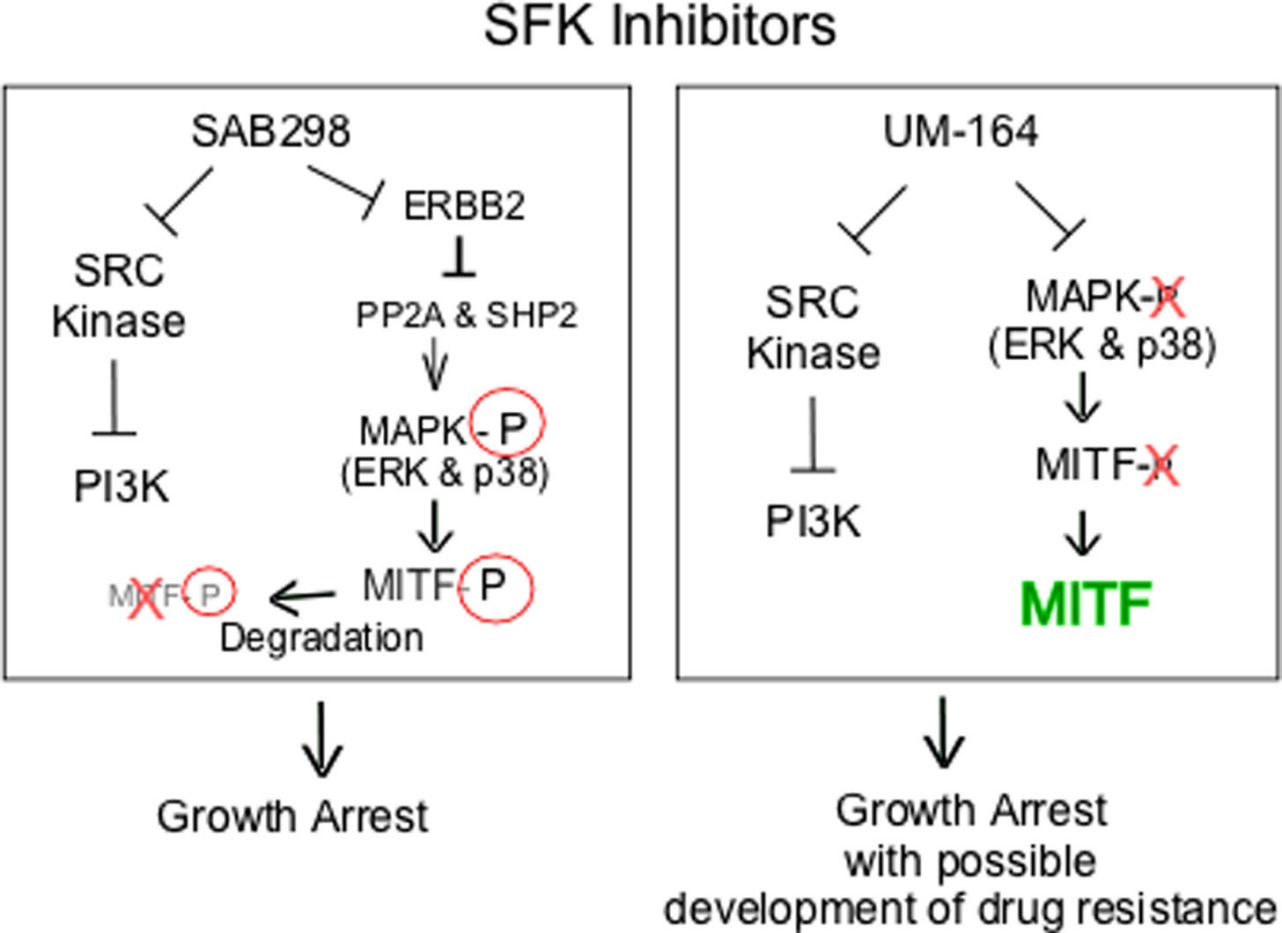
Schematic representation of intracellular signaling induced by SAB298 and UM-164 The figure shows SAB298 and UM-164 common inhibition of SFK and PI3K activities (_┴_) that lead to growth arrest but opposite effects on MITF (down- versus up-regulation) that may contribute to long term response to the drug, such as development of resistance in the presence of high levels of MITF.

We explored the long term effects of SAB298. The substance induced caspase activity and apoptosis, regardless of p53, MYC, p27KIP and cyclin D1 expression (data not shown). On the other hand, we identified in about 57% of the cell lines, a potent and unique SAB298 function of downregulating MITF, a lineage-transcription factor for melanocytes and melanomas. We demonstrated that downregulation of MITF is the consequence of a known pathway, in which MAPK activation phosphorylates the transcription factor and targets it to degradation [27]. In contrast, UM-164 inhibited ERK and caused activation of MITF, an effect that may reduce the long term impact on cell proliferation and contribute to the development of drug resistance as described for BRAF- and MEK inhibitors [28]. Analysis of biopsies from *BRAF*^*V600E*^ melanoma patients following relapse with vemurafenib, or combination of dabrafenib and trametinib revealed upregulation of several lineage-specific transcription factors including MITF [29]. The increased levels of MITF in response to inhibition of MAPK was observed within the first two weeks of treatment [30]. Furthermore, the melanoma cells from tumor-bearing mice treated with vemurafenib were more tolerant to BRAF inhibition than cells isolated from untreated tumors [30], and the protease inhibitor nelfinavir mesylate suppressed MITF expression and sensitizes BRAF and NRAS mutant melanoma to MAPK inhibitor treatment. In another study, *in vitro* and *in vivo* studies with the CH6868398, demonstrated that reduction of MITF levels increased the response to the BRAF inhibitor PLX4720 [31]. Altogether, our data show that one possible cause for resistance to SRC-inhibitors is the increase in MITF, a problem that is not reproduced by SAB298 (summarized in Figure 10).

## MATERIALS AND METHODS

### SAB298 and other SRC-kinase inhibitors

SAB298 was synthesized in six steps according to the procedures outlined in WO 2018/049127 patent application (examples 3, 15, 17, 44, 45 and 46). Its activity was compared to UM-164 (Sigma-Aldrich, St. Louis, MO), dasatinib (BMS 354825), bosutinib (SKI-606), saracatinib, SU6656, sapitinib (AZD8931), ralimetinib (LY2228820), dacomitinib, lifirafenib (BGB-283) and SCH772984 (all from Selleckchem, Houston, TX), okadaic acid (MilliporeSigma, St. Louis, MO), trametinib (LC Laboratories, Woburn), and imatinib (NCI).

### Growth responses and apoptosis

SFK-inhibitors were tested on a panel of NCI-60 cell lines (Supplementary Table 1) and 30 melanoma cell lines and NBMEL, i.e., normal human melanocytes isolated from a newborn foreskin (Table 1 and Supplementary Table 2). The Yale melanoma cohort designated YU originated from tumors excised to improve patient quality of life and used with participants’ informed consent according to Health Insurance Portability and Accountability Act (HIPAA) regulations with Human Investigative Committee protocol.

The melanoma cells were grown in OptiMEM (Invitrogen, Carlsbad, CA) supplemented with 5% fetal calf serum and antibiotics. The normal human melanocytes (NBMEL) were grown in medium supplemented with bFGF, IBMX and dbcAMP [32]. Most of the Yale melanoma cell lines were characterized by next-generation sequencing [7, 33] (Supplementary Table 2).

Cell proliferation was measured with the CellTiter-Glo^®^ Luminescent Cell Viability Assay (Promega Corporation, Madison, WI). Melanoma cells were seeded in 96-well plates in triplicate or quadruplet wells, with increasing concentrations of kinase inhibitors for 72 hrs. The rate of proliferation was also tested after knockdown of different SRC-family kinases (SFK) with hairpin lentivirus shRNA as indicated. The IC_50_ (the dose that elicits 50% inhibition compared to vehicle control) and AUC (area under the curve) were calculated from the slope of the drug response by linear interpolation employing GraphPad Prism 7 software [32]. The statistical significance of synergism of response to drug combinations was evaluated by following Chou and Talalay algorithm [8, 9]. See also details in Supplementary Material.

The rate of apoptosis was measured using the Dead Cell Apoptosis Kit with Alexa Fluor^®^ 488 annexin V and propidium iodide (Invitrogen, V13241). Phosphatidylserine was visualized by flow cytometry following the manufacturer’s instructions. The assay was done in response to SAB298 (0.5 µM and 1 µM, 24 hrs), compared to DMSO (negative control) or camptothecin (10 µM), as a positive control. In addition, we used the 96-well colorimetric Caspase 3 Assay kit (Millipore Sigma) to measure caspase activity in response to increasing concentrations of SAB298, and western blotting for apoptotic markers, such as cleaved PARP.

### Screening of SAB298 cellular targets

The effect of SAB298 on a panel of 36 recombinant kinases, was screed with the radioisotope filter binding assay [10, 26] employing 10 concentrations of the compound with 10 µM ATP (Reaction Biology Corporation, Malvern, PA). In addition, we tested the endogenous kinome response to SAB298 (0.05 µM, 0.5 µM and 5 µM) in lysates from two melanoma cell lines (YUSIV and YUSIK) employing the KiNativ^®^ platform [34] (ActivX Biosciences Inc., La Jolla, CA). KiNativ^®^ is a robust, high performance mass spectrometry (LC-MS^2^) assay that uses biotinylated probes to measure relevant changes in the affinity to the ATP-binding sites of 205 native kinases as a function of cellular context [34]. We also validated SAB298 effect on the activity of SRC-family kinases by probing for phosphorylated Y416, the marker of activated SFKs, with anti-IEDNEpYTAR antibodies (also known as Y419, #2101, Cell Signaling Technology, Beverly, MA). In some cases the cells were incubated overnight with 20 µM proteasome inhibitor MG-132 (CAS 133407-82-6, Millipore Sigma, Burlington, MA) to rescue degradation.

### SFKs-knockdown with short hairpin RNA (shRNA)

We used puromycin-bearing lentiviral vectors pLKO.1 shRNA targeted to SRC, YES, LYN and FYN to test the effects of downregulation of specific kinases on cell proliferation and signal transduction, employing empty vector SHC001 as a negative control (MISSION, Sigma-Aldrich, Supplementary Table 4). The plasmids were packaged in lentiviral vectors with ViraPower™ Lentiviral Packaging Mix kit (Thermo Fisher, cat #K497500), or with two packaging vectors psPAX2 and pMD2G, and transfected into 293T cells. The medium was collected and filtered with Millex-GV 33 mm PVDF filter (Millipore SLGV033RS) and then concentrated with Amicon Ultra-15 centrifugal filters (Millipore UFC910024). Melanoma cells were infected with the lentiviruses, selected with puromycin for two days, and three days later collected and processed for western blotting. In addition, the knockdown cells were seeded in 96-well plates and tested for cell proliferation in the absence and presence of puromycin with the CellTiter-Glo^®^ Luminescent Cell Viability Assay, as described above.

### Western blotting and antibodies

We used standard techniques to identify changes in intracellular signaling in response to various treatments [32]. Briefly, melanoma cells were incubated with test compounds, using DMSO as a control, collected by scraping on ice, and lysed in RIPA buffer supplemented with protease and phosphatase inhibitors (MA #78428, and #78425, respectively, Thermo Fisher Scientific, Waltham, MA). Protein concentrations were measured with the BioRad kit (Bio-Rad Laboratories, Hercules, CA), SDS sample buffer was added, extracts were heated at 95°C, sonicated and centrifuged. Cell extracts (20 µg/lane) were fractionated in 3%–8% or 4–12 % tris-acetate gel (NP0006, NuPAGE Life Technologies) [32]. We probed with the following primary antibodies: phospho-SRC Tyr416 (CST #2101S), pSRC Tyr527 (#2105), SRC (#2108), pPI3K p85 Y458/p55 Y199 (#4228), LYN (C13F9, #2796), FYN (#4023), phospho-MEK1/2 pSer217/221 (#9121), MEK1/2, phospho-ERK2 pThr202/Tyr204 (#9101), ERK1/2 (#9107), phospho-p38 kinase T180/Y182 (#9216), p38 kinase (#9212), PP2A (#2259), pSHP2 Y542 (#3751), SHP2 (D50F2, #3397), ERBB2 (D8F12 XP(R), #4290), phospho-ERBB2 Y1196 (D66B7 #6942), MYC (D84C12, #5605); GAPDH (14C10, #2118), all from Cell Signaling Technology, Beverly, MA; phospho-PP2A Y307 (AF3989), and MITF (clone D5, #AF5769) from R&D Systems, Inc., Minneapolis, MN; p27KIP1 (#10241) and YES (#610375) from BD Biosciences, San Jose, CA; Cyclin D1 (#04-221) and β-actin (mouse mAb A5316) from MilliporeSigma, St. Louis, MO; PI3K Antibody (p85 alpha, NSJ R30480) from Bioreagents, 9921 Carmel Mountain Rd #352, San Diego, CA. All antibodies were used at the concentrations recommended by the manufacturers.

### *In vivo* mouse studies

All procedures involving animals were approved by the Institutional Animal Care and Use Committee of Yale University. YUSIK melanoma cells were grown to 80% confluency in 150 cm^2^ flasks, detached by trypsinization, washed with PBS, and cell pellets were resuspended in 1:1 mixture of PBS and matrigel (BD Bioscience Cat #354234) to sustain cell viability. The cells (4 × 10^6^/100 µl) were injected subcutaneously in the back flank of six weeks old female athymic Nude-Foxn1nu (nu/nu) mice (Charles River Laboratories, Wilmington, MA), and tumors were measuring daily with a caliper (length, width, and depth). When tumors reached palpable size (between 25–40 mm^3^), the animals were randomized by tumor size and body weigh into two groups and were daily injected intraperitoneally with SAB298 (batch #AU0588-91, Sabila Biosciences LLC, 5 Overlook Road, New City, New York, 10956) or vehicle as controls (*N* = 6/each). The injections contained SAB298 (0.5% W/V) in a solution of PBS (30%), DMSO (5%), DMA (5%), PEG400 (20%), and PG (40%) to assure solubility. The vehicle contained the same reagents but without SAB298.

The tumors size and body weight were measured daily and the mice were checked for clinical signs of toxicity, such as lethargy, neurological symptoms, diarrhea, discharges, morbidity, piloerection and weight loss (>20%). None of them or any other abnormal indication were observed.

## SUPPLEMENTARY MATERIALS FIGURES AND TABLES





## Abbreviations

SFK: SRC family kinases
bFGF: basic fibroblast Growth factor
IBMX: isobutyl methyl xanthine
dbcAMP: dibutyryl cyclic AMP
DMSO: dimethyl sulfoxide
DMA: dimethyl acetamide
PG: propylene glycol
