# Supplementary material for: A novel anti-melanoma SRC-family kinase inhibitor
Source: Oncotarget. 2019 Mar 19;10(23):2237–51. doi: 10.18632/oncotarget.26787 (PMC6481345; doi:10.18632/oncotarget.26787)
Supplement: Supplementary file 1 [file oncotarget-10-2237-s001.pdf]

A novel anti-melanoma src-family kinase inhibitor

SUPPLEMENTARY MATERIALS

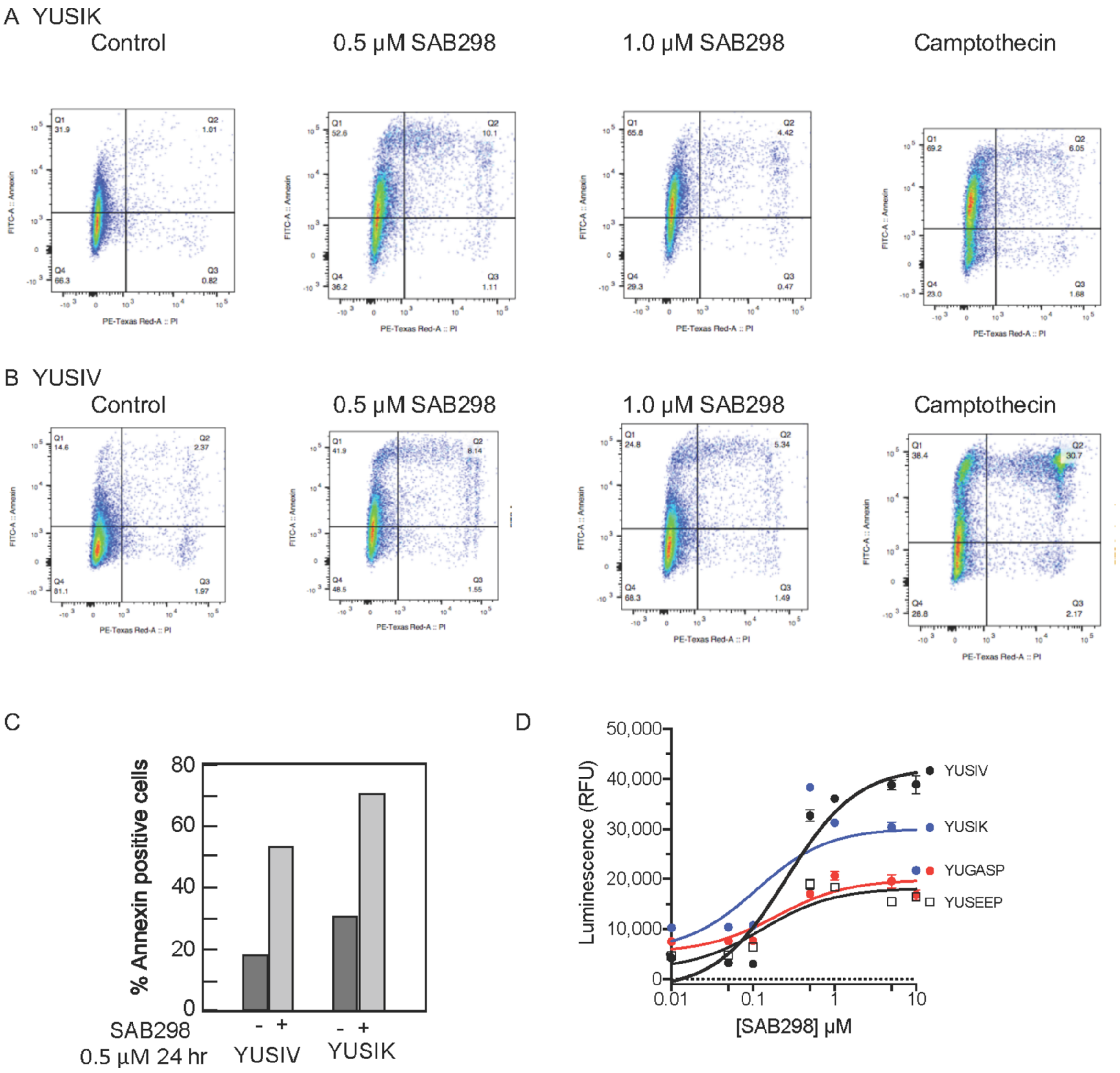

Supplementary Figure 1: Apoptosis in response to SAB298. The figure shows Annexin V staining in YUSIK (**A**) and YUSIV (**B**) melanoma cells treated for 24 hrs with SAB298 (0.5 and 1 µM). (**C**) A bar plot showing the differential levels of annexin positive cells in response to SAB298. (**D**) Caspase activity in response to SAB298 of four different cell lines (YUSIV, YUSIK, YUGASP and YUSEEP).

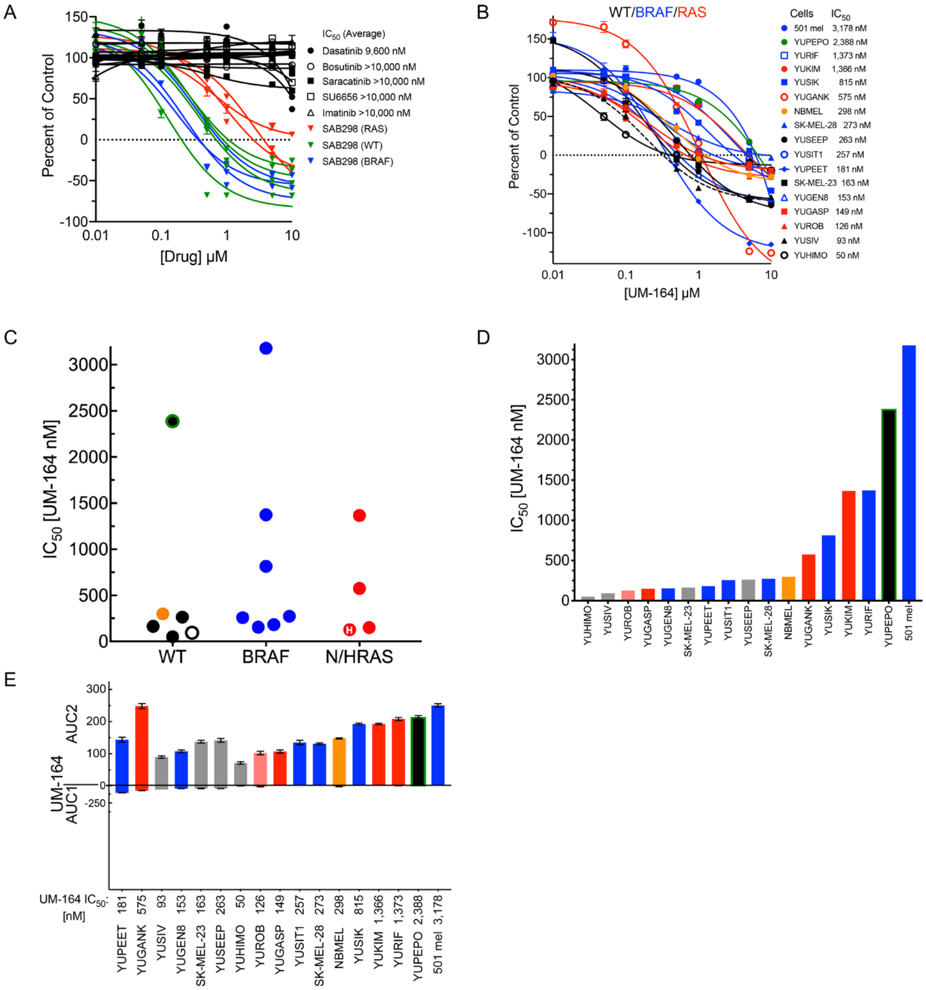

Supplementary Figure 2: Cell proliferation in response to clinically-relevant SFK-inhibitors. (**A**) Black curves show dose response curves, each representing an average of several melanoma cell lines as follows: dasatinib (YUSIV, YUDOSO, YUHIMO), bosutinib (YUSIV, YUSIK, YUGASP, YUSOC, YUROB, 501 mel), saracatinib (YUSIV, SK-MEL-28, YUGASP), SU6656 (YUSIV, YUSIK) and imatinib (YUSIV, YUDOSO, YUHIMO). To highlight the differences we included representative cell lines response to SAB298 taken from Figure 2. Green: (WT/WT) YUHEF, YUSIV, YUHIMO, YUSEEP; Blue: (BRAFV600E/K) YUGEN8, YUSIK, YUZEST; Red: (NRASQ61R/L/K), YUTICA, YROB, YUGASP. Growth response to UM-164 are presented in (**B**–**E**) as follows. (B) Growth curves of individual melanoma cell lines; (C) Aligned dot-plot of IC50 values; (D) Bar graph of increasing levels of IC50; and (E) AUC (Area Under the Curve), plotted to the same scale as Figure 2E for comparison. Orange indicate NBMEL, and Grey bars melanoma with fusion genes.

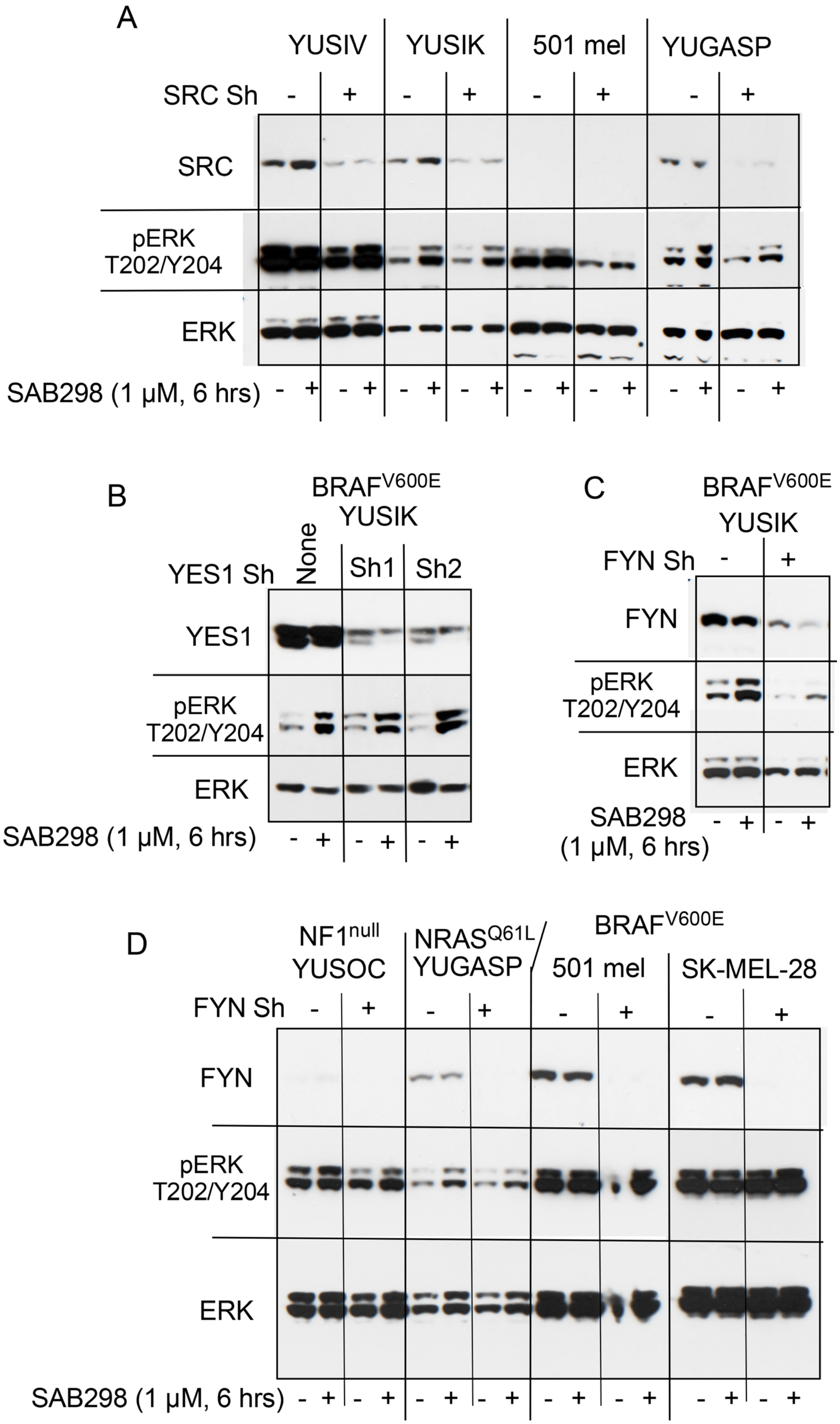

Supplementary Figure 3: Downregulation of SFK does not Affect ERK Phosphorylation. Melanoma cells were infected with SFK shRNA as indicated (described in Figure 5) and the cells were treated with SAB298 (1 µM) for 6 hrs. Probing with SFK and pERK show that downregulation of SRC, YES, and FYN does not eliminate pERK induction in response to SAB298.

Supplementary Table 1: Cellular activity of SAB298 in a Panel of NCI-60 cancer cell lines*

Cell line	GI_{50} (nM)	LC_{50} (nM)	TI
**Melanomas**			
M14	21	105	5
SK-MEL-28	88	>100,000	>1136
MDA-MB-435	103	618	6
LOX IMVI	170	4,555	26.8
UACC-62	182	872	4.8
SK-MEL-2	212	19,450	91.7
SK-MEL-5	251	2,675	10.6
UACC-257	374	93,500	250
MALME-3M	550	>100,000	>181
**Leukemia**			
CCRF-CEM	61	>100,000	>1,640
MOLT-4	32	>100,000	>3,125
SR	38	>100,000	>2,631
**NSCLC**			
HOP-62	227	23,550	104
NCI-1460	29	551	19
**Colon Cancer**			
HCT-116	134	6,520	49
KM12	181	79,300	438
**CNS Cancer**			
SF-268	38	>100,000	>2,631
**Breast Cancer**			
MCF7	138	52,980	384

*The results show five dose response curve, each value is an average of 2 experiments.
GI_{50} is the concentration of the drug causing 50% growth inhibition; LC_{50} is the cytotoxic concentration due to 50% reduction in measured protein; and TI is LC_{50}/GI_{50}.

Supplementary Table 2: Description of Melanoma Cell lines. See Supplementary_Table_2

Supplemntary Table 3: Radioisotope filter activity assay Tests for SAB298 target protein

Kinase	IC_{50} (nM)*
YES1	0.7
BLK	6.5
LCK	7.8
FGR	7.8
HCK	16.3
FYN	21.7
BRK	35.5
FLT3	71
BTK	78
LYN	137
CSK	139
SRC	269
ARAF	1,200
ABL2	2,560
RAF1	2,800
ABL1	4140
ERBB2	8,864
BRAF	>10,000
IGF1R	>10,000
CDK4/Cyclin D1	>10,000
WEE1	>10,000

*These are results from 10 point dose response test employed with 10 μM ATP.

Supplementary Table 4: Lentiviral vectors MISSION pLKO.1 puromycin bearing SFK shRNA used to test the effect of specific SFK downregulation on cell proliferation

Gene symbol	Designation	Region	Sequence
SRC	TRCN0000195339	3′UTR	CCGGCATCCTCAGGAACCAACAATTCTCGAGAATTGTTGGTTC CTGAGGATGTTTTTTG
YES	TRCN0000001611	CDS	CCGGACCACGAAAGTAGCAATCAAACTCGAGTTTGATTGCTAC TTTCGTGGTTTTTT
FYN	TRCN0000003099	CDS	CCGGGCCTATTCACTTTCTATCCGTCTCGAGACGGATAGAAAG TGAATAGGCTTTTT
LYN	TRCN0000230901	CDS	CCGGGAGTGACGATGGAGTAGATTTCTCGAGAAATCTACTCCA TCGTCACTCTTTTTG

The short hairpin RNA Lentiviral vectors were from The Functional Genomics Shared Resource Core of the Yale Cancer Center, David A Calderwood and Ben E. Turk, directors.

SUPPLEMENTARY METHOD

Statistical method for synergistic effects of drug combination

We used the median-effect equation derived from the mass-action law principle [1, 2] to quantify the drug combination effect. Given a set of measurements representing the dose-effect of a drug, we can characterize this relationship by fitting the median-effect equation (see Eq. 1) to the measurements to estimate two parameters: 1) D_m, which represents the required dose to achieve the median effect (i.e. equivalent to IC_{50}, ED_{50}) and 2) m, which represents the slope of the regression line fitted to the measurements when they are plotted using $\log\left(\frac{f_a}{1-f_a}\right)$ as y-axis and log(D) as x-axis. The slope m determines the shape of the dose-effect curve which can be hyperbolic when m = 1, sigmoidal for m > 1 and negative sigmoidal when m < 1 [3]. The variables f_a and D represent the effect of the drug on a scale from 0 to 1 and the drug dosage respectively.

$$\frac{f_a}{1-f_a}=\left[\frac{D}{D_m}\right]^m \quad \text{(Equation 1)}$$

In other words, we first plot the dose-effect measurements using the $\log\left(\frac{f_a}{1-f_a}\right)$ as the y-axis and log(D) as the x-axis, and then we fit a regression line to the data in order to estimate the two parameters D_m and m.

After estimating both D_m and m values using the dose-effect measurements of a drug, we can rearrange Equation 1 to compute the dosages D_x for various effect levels f_a where x refers to the fractional effect (i.e. x = 50 refers to median-effect where $f_a = 1 - f_a = 0.5$). Hence, when x = 50, $D_{50} = D_m$ (substitute in Equation 2 for verification).

$$D_x=\left(\frac{f_a}{1-f_a}\right)^{\frac{1}{m}} D_m \quad \text{(Equation 2)}$$

To determine the combined effect of two drugs $(D)_1$ and $(D)_2$, we compute the combination index (CI) based on the median-effect equation, which quantifies the degree of drug interaction where CI <1 refers to synergistic relation, CI = 1 additive relation, and CI > 1 antagonistic relation [2]. Generally, two cases are considered when studying the combined effect of two drugs: 1) the first case is when both drugs are considered mutually exclusive, and 2) the second when they are mutually non-exclusive.

Case 1: Two drugs are mutually exclusive

When two drugs are considered mutually exclusive, the combined effect or their CI value can be computed using Equation 3

$$CI=\frac{(D)_1}{(D_x)_1}+\frac{(D)_2}{(D_x)_2} \quad \text{(Equation 3)}$$

where $(D_x)_1$ refers to computed dosage of the first drug $(D)_1$ for fractional effect (i.e. for predefined effect level f_a). Similarly, where $(D_x)_2$ refers to computed dosage of the second drug $(D)_2$ for x fractional effect (i.e. for predefined effect level f_a).

Case 2: Two drugs are mutually non-exclusive

When two drugs are considered mutually non-exclusive, the combined effect or their CI value can be computed using Equation 4

$$CI=\frac{(D)_1}{(D_x)_1}+\frac{(D)_2}{(D_x)_2}+\frac{(D)_1(D)_2}{(D_x)_1(D_x)_2} \quad \text{(Equation 4)}$$

As it can be noted the difference between Equations 3 and 4 is the added multiplicative term $\frac{(D)_1(D)_2}{(D_x)_1(D_x)_2}$.

To measure the effect of the drug in an experiment, we first average the experimental numbers across the three trials at T72 (i.e. after 72 hours). Then at each dose level, we take the ratio of the counts corresponding to a particular drug level over the counts in the control (i.e. no drug condition). We then transformed the ratios to a scale from 0 to 1, in which higher values on the transformed scale indicates lower counts in comparison to the control (no drug case). This allowed us to measure the observed effect f_a for the two drugs and their combination in the three experiments.

In the case of drug combination, we had a non-constant ratio combination such that we fix the dosage of $(D)_2$ SAB298 and we vary the dosage of $(D)_1$ Selumetinib (MEKi) as before. All dose levels reported are in µM.

To measure the combined effect of the two drugs, we computed the combination index (CI) based on the median-effect equation as described before [1, 2]. We used CompuSyn [3] and we developed a Python script implementing the median-effect equations to compute CI index for the mutually exclusive case and the mutually non-exclusive case. We performed the analysis in two variations: 1) the first included all measurements of dose-effect in our experiments where 0 observed effect levels were coded as 1E-6 and 2) the second omitted measurements having observed effect levels equal to 0 (i.e. were considered as outliers). We refer to both variations in the text as Variation 1 and 2 respectively.

RESULTS

Table 1: Estimates of the parameters obtained by fitting median-effect equation to dose-effect measurements of each drug in each experiment

	$(D)_1$:Selumetinib (MEKi)			$(D)_2$:SAB298		
**Experiment**	D_m	**m**	**r**	D_m	m	r
YUSIK BRAF^{V600E}	0.71970	0.76339 ± 0.16704	0.89824	0.86391	2.85159 ± 0.67642	0.88342
501 mel BRAF^{V600E}	5.72031	1.13636 ± 0.71771	0.57788	7.15045	1.52370 ± 0.93746	0.58796
YUROB HRAS^{Q61K}	6.26267	0.31358 ± 0.04218	0.95763	5.17854	1.66622 ± 0.92060	0.62915

r represents the correlation coefficient. Measurements having 0 effect were represented by 1E-6 (Variation 1).

Table 2: Estimates of the parameters obtained by fitting median-effect equation to dose-effect measurements of each drug in each experiment

	$(D)_1$:Selumetinib (MEKi)			$(D)_2$:SAB298		
**Experiment**	D_m	**m**	**r**	D_m	**m**	**r**
YUSIK BRAF V600E	0.71970	0.76339 ± 0.16704	0.89824	0.15499	1.12217 ± 0.24947	0.93321
501 mel BRAF V600E	6.25618	0.52099 ± 0.09853	0.93533	3.86105	0.43436 ± 0.08859	0.94290
YUROB HRAS Q61K	6.26267	0.31358 ± 0.04218	0.95763	1.86331	0.59760 ± 0.07865	0.97499

r represents the correlation coefficient. Measurements having 0 effect were omitted (Variation 2).

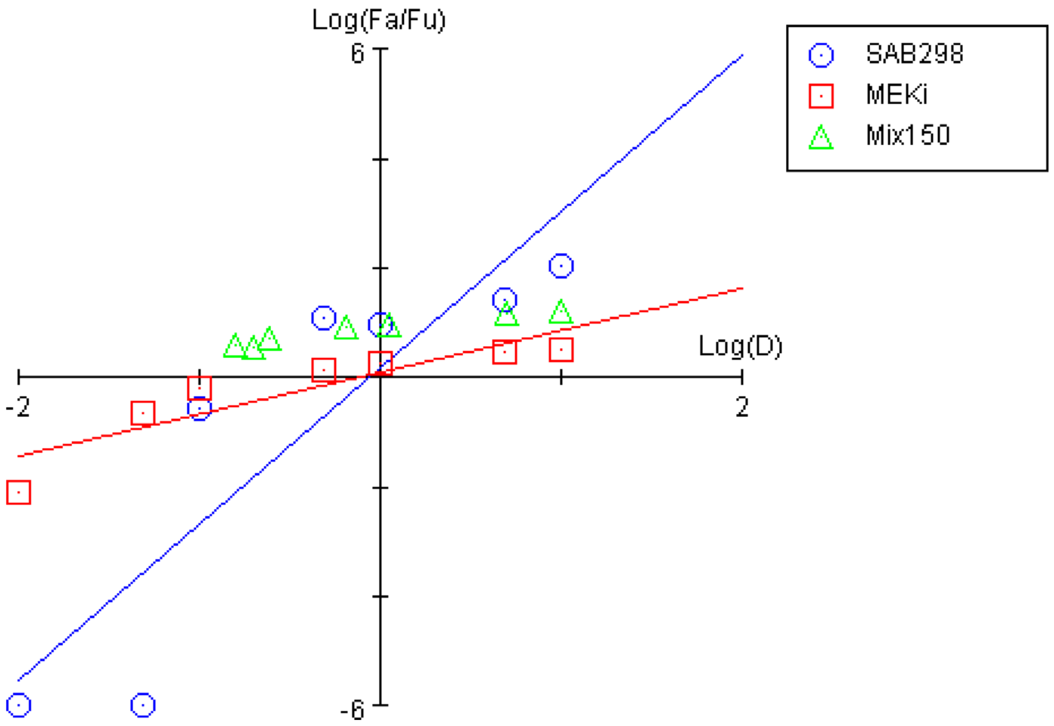

Figure 1: Median-effect plot for YUSIK BRAF V600E experiment. Analysis using data from Variation 1.

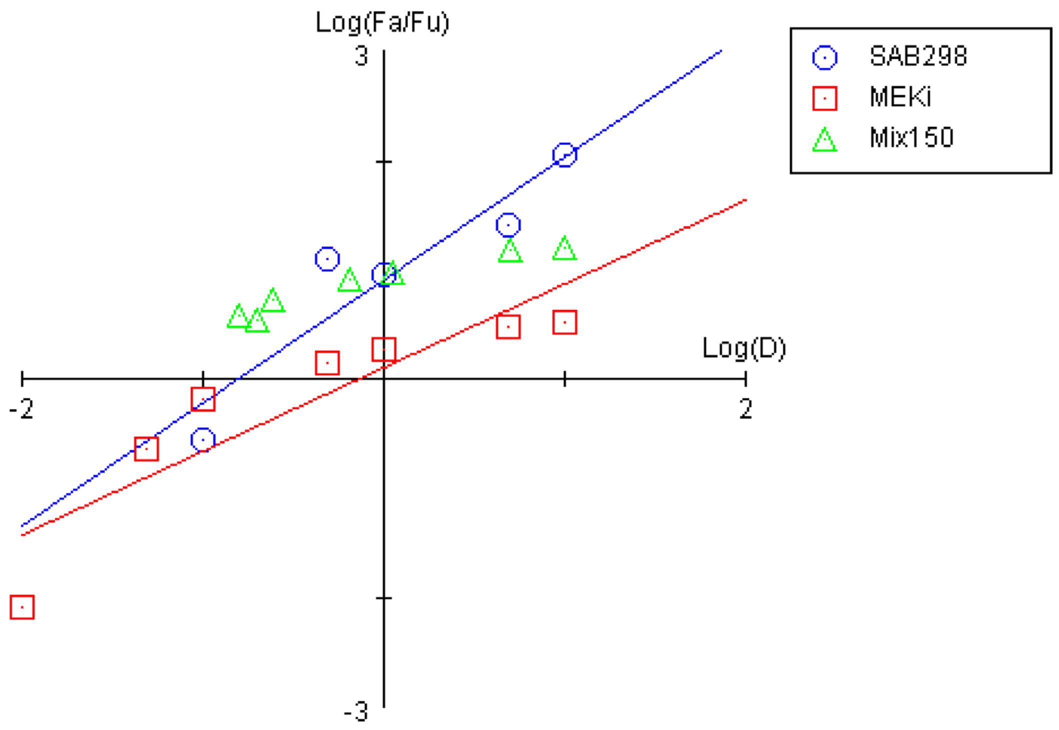

Figure 2: Median-effect plot for YUSIK BRAF V600E experiment. Analysis using data from Variation 2

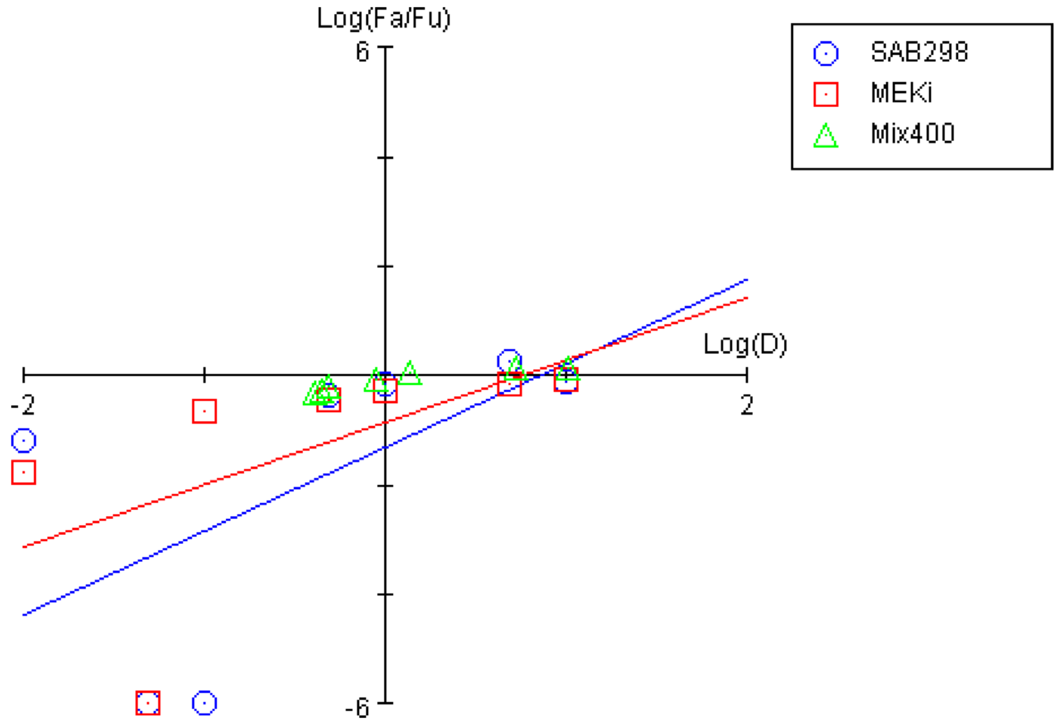

Figure 3: Median-effect plot for 501 mel BRAF V600E experiment. Analysis using data from Variation 1.

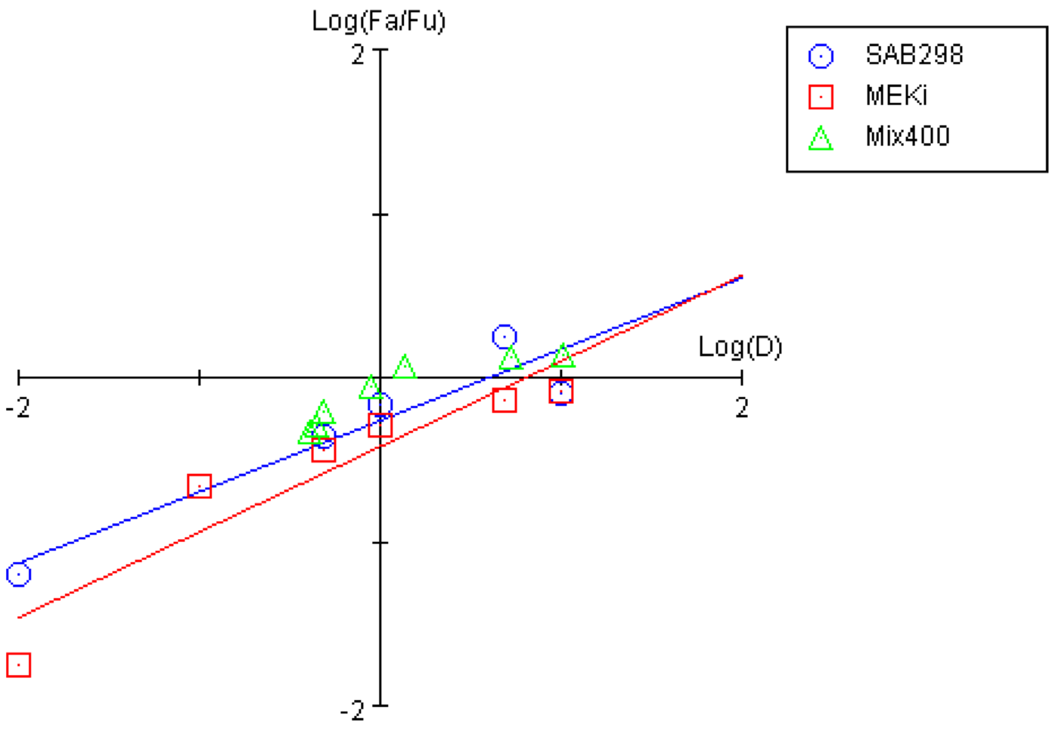

Figure 4: Median-effect plot for 501 mel BRAF V600E experiment. Analysis using data from Variation 2.

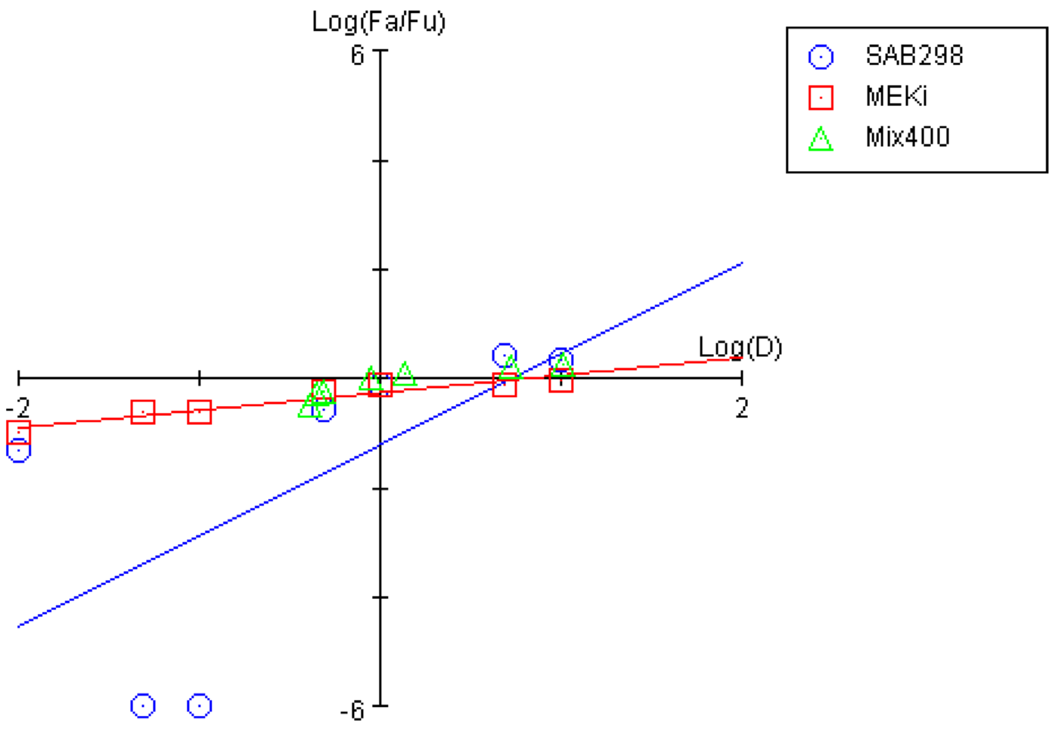

Figure 5: Median-effect plot for YUROB HRAS Q61K experiment. Analysis using data from Variation 1.

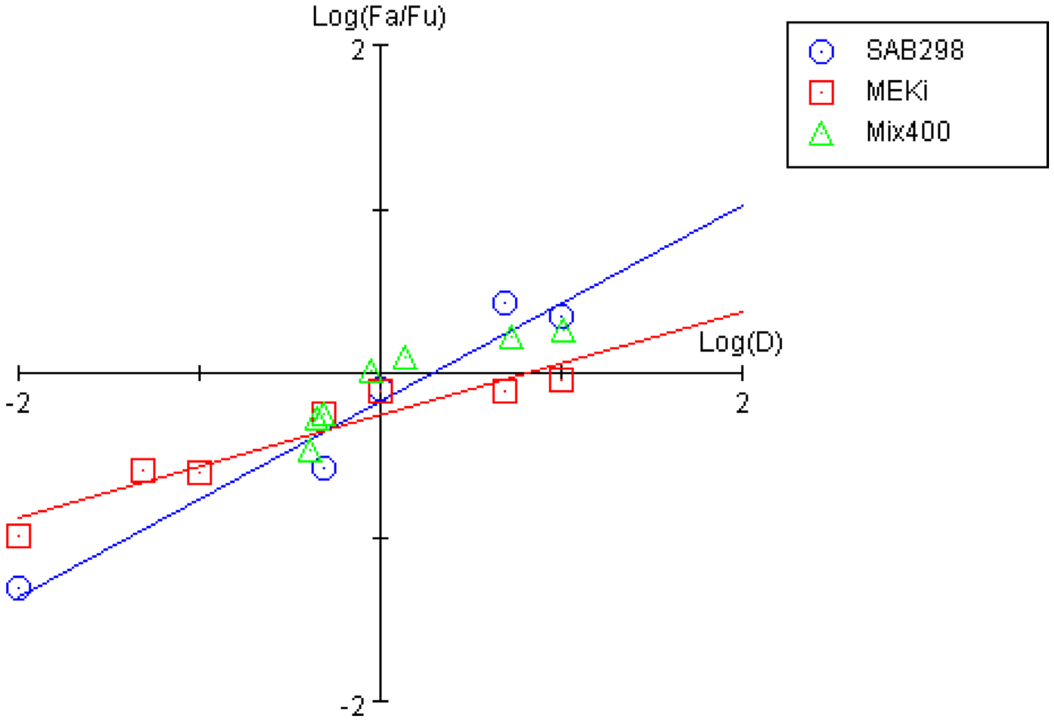

Figure 6: Median-effect plot for YUROB HRAS Q61K experiment. Analysis using data from Variation 2

Table 3: CI values at different measured effect levels for 501 mel BRAF V600E experiment

$(D)_1$:Selumetinib (MEKi)	$(D)_2$:SAB298	Mode	Effect level	CI
10	0.4	mutually exclusive	0.5826	1.3488
5	0.4	mutually exclusive	0.5772	0.7101
1	0.4	mutually exclusive	0.5402	0.202
0.5	0.4	mutually exclusive	0.4749	0.1552
0.1	0.4	mutually exclusive	0.3896	0.1011
0.05	0.4	mutually exclusive	0.3454	0.1004
0.01	0.4	mutually exclusive	0.3195	0.0953
10	0.4	mutually non-exclusive	0.5826	1.4074
5	0.4	mutually non-exclusive	0.5772	0.7404
1	0.4	mutually non-exclusive	0.5402	0.2097
0.5	0.4	mutually non-exclusive	0.4749	0.1609
0.1	0.4	mutually non-exclusive	0.3896	0.103
0.05	0.4	mutually non-exclusive	0.3454	0.1017
0.01	0.4	mutually non-exclusive	0.3195	0.0956

CI values were computed for both mutually exclusive and mutually non-exclusive assumptions. Analysis using data from Variation 1.

Table 4: CI values at different measured effect levels for 501 mel BRAF V600E experiment

$(D)_1$:Selumetinib (MEKi)	$(D)_2$:SAB298	Mode	Effect level	CI
10	0.4	mutually exclusive	0.58255	0.8912322
5	0.4	mutually exclusive	0.57724	0.4901533
1	0.4	mutually exclusive	0.54018	0.1888355
0.5	0.4	mutually exclusive	0.47494	0.2274085
0.1	0.4	mutually exclusive	0.38959	0.3291328
0.05	0.4	mutually exclusive	0.34541	0.4786346
0.01	0.4	mutually exclusive	0.3195	0.5974465
10	0.4	mutually non-exclusive	0.58255	0.9317877
5	0.4	mutually non-exclusive	0.57724	0.5123855
1	0.4	mutually non-exclusive	0.54018	0.197225
0.5	0.4	mutually non-exclusive	0.47494	0.2400545
0.1	0.4	mutually non-exclusive	0.38959	0.3401565
0.05	0.4	mutually non-exclusive	0.34541	0.4909399
0.01	0.4	mutually non-exclusive	0.3195	0.6014761

CI values were computed for both mutually exclusive and mutually non-exclusive assumptions. Analysis using data from Variation 2.

Table 5: CI values at different measured effect levels for YUROB HRAS Q61K experiment

$(D)_1$:Selumetinib (MEKi)	$(D)_2$:SAB298	Mode	Effect level	CI
10	0.4	mutually exclusive	0.6503	0.274
5	0.4	mutually exclusive	0.6317	0.1989
1	0.4	mutually exclusive	0.5616	0.1391
0.5	0.4	mutually exclusive	0.5149	0.1405
0.1	0.4	mutually exclusive	0.3659	0.1997
0.05	0.4	mutually exclusive	0.349	0.1706
0.01	0.4	mutually exclusive	0.2592	0.1905
10	0.4	mutually non-exclusive	0.6503	0.2858
5	0.4	mutually non-exclusive	0.6317	0.2068
1	0.4	mutually non-exclusive	0.5616	0.1439
0.5	0.4	mutually non-exclusive	0.5149	0.1454
0.1	0.4	mutually non-exclusive	0.3659	0.2096
0.05	0.4	mutually non-exclusive	0.349	0.1772
0.01	0.4	mutually non-exclusive	0.2592	0.1971

CI values were computed for both mutually exclusive and mutually non-exclusive assumptions. Analysis using data from Variation 1.

Table 6: CI values at different measured effect levels for YUROB HRAS Q61K experiment

$(D)_1$:Selumetinib (MEKi)	$(D)_2$:SAB298	Mode	Effect level	CI
10	0.4	mutually exclusive	0.6503	0.2968
5	0.4	mutually exclusive	0.6317	0.23
1	0.4	mutually exclusive	0.5616	0.2144
0.5	0.4	mutually exclusive	0.5149	0.2603
0.1	0.4	mutually exclusive	0.3659	0.6311
0.05	0.4	mutually exclusive	0.349	0.6677
0.01	0.4	mutually exclusive	0.2592	1.2896
10	0.4	mutually non-exclusive	0.6503	0.3136
5	0.4	mutually non-exclusive	0.6317	0.2425
1	0.4	mutually non-exclusive	0.5616	0.2247
0.5	0.4	mutually non-exclusive	0.5149	0.2731
0.1	0.4	mutually non-exclusive	0.3659	0.6808
0.05	0.4	mutually non-exclusive	0.349	0.7033
0.01	0.4	mutually non-exclusive	0.2592	1.3461

CI values were computed for both mutually exclusive and mutually non-exclusive assumptions. Analysis using data from Variation 2.

Table 7: CI values at different measured effect levels for YUSIK BRAF V600E experiment

$(D)_1$:Selumetinib (MEKi)	$(D)_2$:SAB298	Mode	Effect level	CI
10	0.15	mutually exclusive	0.9413	0.4322
5	0.15	mutually exclusive	0.9382	0.264
1	0.15	mutually exclusive	0.9045	0.152
0.5	0.15	mutually exclusive	0.8943	0.1245
0.1	0.15	mutually exclusive	0.8432	0.1116
0.05	0.15	mutually exclusive	0.7794	0.1248
0.01	0.15	mutually exclusive	0.7983	0.1095
10	0.15	mutually non-exclusive	0.9413	0.4562
5	0.15	mutually non-exclusive	0.9382	0.2772
1	0.15	mutually non-exclusive	0.9045	0.1578
0.5	0.15	mutually non-exclusive	0.8943	0.128
0.1	0.15	mutually non-exclusive	0.8432	0.1131
0.05	0.15	mutually non-exclusive	0.7794	0.1263
0.01	0.15	mutually non-exclusive	0.7983	0.1097

CI values were computed for both mutually exclusive and mutually non-exclusive assumptions. Analysis using data from Variation 1.

Table 8: CI values at different measured effect levels for YUSIK BRAF V600E experiment

$(D)_1$:Selumetinib (MEKi)	$(D)_2$:SAB298	Mode	Effect level	CI
10	0.15	mutually exclusive	0.9413	0.4482
5	0.15	mutually exclusive	0.9382	0.2829
1	0.15	mutually exclusive	0.9045	0.2036
0.5	0.15	mutually exclusive	0.8943	0.1868
0.1	0.15	mutually exclusive	0.8432	0.2315
0.05	0.15	mutually exclusive	0.7794	0.3276
0.01	0.15	mutually exclusive	0.7983	0.2863
10	0.15	mutually non-exclusive	0.9413	0.4781
5	0.15	mutually non-exclusive	0.9382	0.2998
1	0.15	mutually non-exclusive	0.9045	0.2131
0.5	0.15	mutually non-exclusive	0.8943	0.1929
0.1	0.15	mutually non-exclusive	0.8432	0.2348
0.05	0.15	mutually non-exclusive	0.7794	0.3317
0.01	0.15	mutually non-exclusive	0.7983	0.287

CI values were computed for both mutually exclusive and mutually non-exclusive assumptions. Analysis using data from Variation 2.

Table 9: CI ranges classification adapted from Chou and Marin [3]

Range of combination index	Description
<0.1	Very strong synergism
0.1–0.3	Strong synergism
0.3–0.7	Synergism
0.7–0.85	Moderate synergism
0.85–0.9	Slight synergism
0.9–1.10	Nearly additive
1.10–1.20	Slight antagonism
1.20–1.45	Moderate antagonism
1.45–3.3	Antagonism
3.3–10	Strong antagonism
>10	Very strong antagonism

REFERENCES

1. Chou TC. Theoretical basis, experimental design and computerized simulation of synergism and antagonism in drug combination studies. Pharmacol Rev. 2006; 58:621–681.
2. Chou TC, Talalay, P. Quantitative analysis of dose-effect relationship: the combined effects of multiple drugs or enzyme inhibitors. Adv Enzyme Regul. 1984; 22:27–55.
3. Chou TC, Martin, N. CompuSyn software for drug combinations and for general dose-effect analysis, and user's guide. ComboSyn, Inc. Paramus, NJ. 2007. http://www.combosyn.com.
