# Supplementary material for: A novel anti-melanoma SRC-family kinase inhibitor
Source: Oncotarget. 2019 Mar 19;10(23):2237–51. doi: 10.18632/oncotarget.26787 (PMC6481345; doi:10.18632/oncotarget.26787)
Supplement: Supplementary file 2 [file oncotarget-10-2237-s002.docx]

**Supplementary Table 2: Decripiotn of Melanoma Cell lines.**

| Cell Line | Tumor Type | SAB298 IC_50_ [nM] | Mutation BRAF-NRAS | Other Oncogen Mutations | References (WES)* |
| --- | --- | --- | --- | --- | --- |
| YUHEF | Sun-exposed | 257 | WT/WT | RAC1 p.P29S; NF1 p.Q853X, NF1 INDEL; SOS1 p.P102S/R398G/G434V; CDKN2A p.A57X/p.P113L; ARID1A p.M618L; ARID1A p.M618L; JARID2 p.D298N; ARID1B p.R1953H; TRRAP p.F3430L | (1) |
| YUTOGS | Sun-exposed | 347 | WT/WT | RAC1 p.P29S; NF1 p.K2535X/R461X; ARID2 p.Q1165X/p.Q1313X; FBXW7 p.501X/p.R578X; ARFGAP1 p.P264L | (2) |
| YUPEPO | Ocular | 264 | WT/WT | GNA11 p.Q209L; KMT2D p.D966V; DNMT1 p.G1058S | Not published |
| YUROL | Sun-exposed | 717 | WT/WT | CDKN2A p.W110X; BRCA2 p.M815V; ARID2 p.I1322F/p.D1521G; SMARCD1 p.P61L; ABCB5 p.R892T | (1) |
| YUCHIME | Sun-exposed | 2,477 | BRAF p.V600K/M | NF1 p.K1714N; MAP3K11 p.L712R; CDKN2A p.P114L; PIK3C2A p.E1587K; PIK3CA p.R230X; TP53 p.E286K; | (1) |
| YURIF | Sun-exposed | 500 | BRAF p.V600K | RAC1 p.P29S; CDKN2A p.R62K; PPP6C p.S307L; PTEN p.E288K, CNOT9 p.P131S | (1) |
| YUKSI | Sun-exposed | 582 | BRAF p.V600K | MAP2K1 p.P124S; MAP2K3 p.N37S; TP53 p.Q317X; ARID3B p.L53F; SMARCA1 p.R306X; SMARCC1 p.P1043S | (1) |
| YUMAC | Sun-exposed | 894 | BRAF p.V600K | BAP1 p.Q335X; PTEN p.E288K/p.E291K/p.G1893A | (2) |
| YUSIT1 | unknown | 238 | BRAF p.V600K | MAP2K2 p.E27K; TP53 p.Q317X; TYRP1 p.P247S; OCA2 p.P114L; SMARCA5 p.L781X; MAP2K2 p.E27K | (2) |
| 501 mel | Sun-exposed | 551 | BRAF p.V600E | CTNNB1 p.S45F/p.D32H; CDKN2A p.L3/p.L37del; TP53 p.C275W; KMT2C p.Y816X; CDC27 p.L254X; MAP2K3 p.Q102X; ARID4B p.L340P; BRCA1 p.Q356R | Not published |
| SK-MEL-28 | Sun-exposed | 812 | BRAF p.V600E | EGFR p.P753S | (3) |
| YULIPPY | Ocular | 111 | BRAF p.V600E | GNA11 p.Q209L | WES failed |
| YUPEET | Sun-exposed | 55 | BRAF p.V600E | HDAC4 p.D840N; KMT2A, p.R862X; SYK p.D428N; FLG, p.R826Q | (1) |
| YUSIK | Sun-exposed | 141 | BRAF p. V600E | MAP2K3 p.P162L; PTEN p.E288K | (2) |
| YUZEST | unknown | 263 | BRAF p.V600E | CDKN2A p.R58X; BRCA1 p.R496C; ATM p.L186I; SMARCA4 p.P1090S | (1) |
| YUGEN8 | unknown | 115 | BRAF p.V600E | SMARCA4 p.N1548D; SMARCC1 p.Q431K; TRRAP p.P1535S; PTEN null | (1) |
| YUCRATE | Acral | 282 | BRAF p.G469A | MAPK6 p.E520K | (2) |
| SK-MEL-23 | unknown | 162 | WT/WT | Not known |  |
| YUSOC | Sun-exposed | 157 | RAF1 p.T491I | NF1 p.W336X/E337K; RAC1 p.P29S; ARID2 p.R314C/p.Q476X; SMARCC1 p.I173V; RASA2 p.R511C/p.Q500X; CDKN2A p.R80X; RB1 p.S842F; EZH2 p.Y646H; | (2) |
| YUTICA | Sun-exposed | 766 | NRAS p.Q61R | NF1 p.P1667L; PTPN11 p.N58S; MAPK9 p.P319S; ARID1A p.Q1493K; ARID2 p.Q490X; GABRA3 p.P57S; BCL2L12 p.P272S; MITF null | (1) |
| YUKIM | Sun-exposed | 561 | NRAS p. Q61R | CTNNB1 p.S33F; CDK11 p.E477V; BRCA2 p.R2799K; PTEN p.E288K; SMARCA4 p.R1135Q/p.A1423V; INSR V1047I | (2) |
| YUGASP | Sun-exposed | 738 | NRAS p.Q61L | NF1 LOH; CDKN2A-loss; FANCA p.P667L; MITF null | (1) |
| YUDOSO | Sun-exposed | 2,359 | NRAS p.Q61K | CDKN2A p.D74A; CDK13 p.Q103R | (2) |
| YUGANK | Sun-exposed | 456 | NRAS p.Q61K | PPP6C p.Q220X/R301C; CDKN2A p.R80X; BRCA1 p.L750F; ARID4A p.N1179I; SMARCA1 p.P840L; SMARCAD1 p.P69L | (2) |
| YUSIV | Sun-exposed | 81 | PDE8A-RAF1 fusion | NF1 p.L626F; BRCA1 p.V772A; PTEN p.E288K; TRRAP p.S722F; PCDHGA1 p.P155L | (1) |
| YUHIMO | Acral | 237 | PDE4DIP-BRAF fusion | CDKN2A loss; CDKN2B loss; TERT Gain; LZTR1 Gain; CRKL Gain, EP300 Gain; SOX10 Gain; SRC Gain | (2) |
| YUFURL | Mucosal | 371 | NRAS p.G12D | PIK3CA p.Q546R; SF3B1 p.R625H; NOTCH1 p.G1039D; YES1 p.Y537C; ARID2 p.T311A; DUSP9 p.R231C; SOX10 p.A361T; SRSF3 p.R64X | Not published |
| YUROB | Sun-exposed | 1,249 | HRAS p.Q61K | ARID2 p.Q1118X; ATM p.G2180E; CTNNB1 p.P52L; ARHGAP5 p.Q874X; AKAP9 p.Q1154X | (2) |
| YUNIS | Acral | 2,577 | HRAS p.Q61K | ARID2 p.K706I; CDK17 p.S463L; AGAP2 p.E438K; PTPRB p.P1503S/Q ; STRN p.T486I; TERT Gain; CCND1 Gain; LZTR1 Gain; CRKL Gain, EP300 Gain; SOX10 Gain | Not published |
| YUSEEP | Acral | 274 | GOLGA4-RAF1 fusion | EWSR1 p.G290E; CDKN2A loss; CDKN2B loss; TERT Gain; CCND1 Gain; LZTR1 Gain; CRKL Gain, EP300 Gain; SOX10 Gain | Not published |
| NBMEL | Normal melanocytes | 968 | WT | None | Not published |

References

1. Krauthammer M, Kong Y, Bacchiocc hi A, Evans P, Pornputtapong N, Wu C, McCusker JP, Ma S, Cheng E, Straub R, Serin M, Bosenberg M, Ariyan S, et al. Exome sequencing identifies recurrent mutations in NF1 and RASopathy genes in sun-exposed melanomas. Nat Genet. 2015; 47:996-1002.

2. Krauthammer M, Kong Y, Ha BH, Evans P, Bacchiocchi A, McCusker JP, Cheng E, Davis MJ, Goh G, Choi M, Ariyan S, Narayan D, Dutton-Regester K, et al. Exome sequencing identifies recurrent somatic RAC1 mutations in melanoma. Nat Genet. 2012; 44:1006-14.

3. ATCC cell lines by gene mutation. ATCC. 2019. Available from <https://www.atcc.org/~/media/PDFs/Culture%20Guides/Cell_Lines_by_Gene_Mutation.ashx>

Our Melanoma Exome Sequencing”  phs000933.v2.p1 is publicly available dbGaP

web page [https://www.ncbi.nlm.nih.gov/projects/gap/cgi-bin/study.cgi?study_id=phs000933.v2.p1](https://na01.safelinks.protection.outlook.com/?url=https%3A%2F%2Fwww.ncbi.nlm.nih.gov%2Fprojects%2Fgap%2Fcgi-bin%2Fstudy.cgi%3Fstudy_id%3Dphs000933.v2.p1&data=02%7C01%7Cruth.halaban%40yale.edu%7C95ae9c5276324cb9f1c608d651888d1b%7Cdd8cbebb21394df8b4114e3e87abeb5c%7C0%7C0%7C636786044269702495&sdata=jMIahcD%2BhNAHOTfpX3y3J6CdkwcgFplPwuH4A9%2BCz8s%3D&reserved=0).
